# Impact of Δ^9^-tetrahydrocannabinol and oxycodone coadministration on measures of antinociception, dependence, circadian activity, and reward in mice

**DOI:** 10.1016/j.jpet.2026.105008

**Published:** 2026-08-14

**Authors:** Richard A. Slivicki, Justin G. Wang, Kayla V. Kucz, Jakayla Folarin Hines, Vy Trinh Tran Nhat, Alexxai V. Kravitz, Meaghan C. Creed, Robert W. Gereau

**Affiliations:** 1 Department of Anesthesiology, Washington University Pain Center, St. Louis, Missouri; 2 Division of Biology and Biomedical Sciences, Neuroscience Graduate Program, St. Louis, Missouri; 3 Department of Psychiatry, Washington University, St. Louis, Missouri; 4 Department of Neuroscience, Washington University, St. Louis, Missouri; 5 Department of Biomedical Engineering, Washington University, St. Louis, Missouri

**Keywords:** Oxycodone, Δ^9^-Tetrahydrocannabinol, Antinociception, Dependence, Reward, Photometry

## Abstract

Oxycodone is commonly prescribed for moderate-to-severe pain. While efficacious, long-term use can result in tolerance, physical dependence, and opioid use disorder. Cannabis and Δ^9^-tetrahydrocannabinol (Δ^9^-THC) have been reported to potentiate oxycodone-induced antinociception in animals and humans. Whether Δ^9^-THC also facilitates unwanted aspects of oxycodone intake, such as tolerance, dependence, and reward at antinociceptive doses, remains unclear. We evaluated the coadministration of Δ^9^-THC and oxycodone on antinociception, dependence, circadian activity, and reward in male and female mice. Oxycodone and Δ^9^-THC produced dose-dependent antinociception in the hotplate assay, with oxycodone showing lower potency in females. Repeated treatment (twice daily for 5 days) produced antinociceptive tolerance. Δ^9^-THC produced a potentiation-like effect on oxycodone-induced antinociception and delayed antinociceptive tolerance. Repeated oxycodone produced physical dependence and alterations in circadian activity, neither of which was exacerbated by cotreatment with Δ^9^-THC. Combination treatment of oxycodone and Δ^9^-THC produced conditioned place preference at doses that did not produce preference when administered alone. Fiber photometry recordings of nucleus accumbens dopamine release showed that Δ^9^-THC potentiated oxycodone-evoked dopamine release at subeffective oxycodone doses, a candidate mechanism for the behavioral reward potentiation. These data indicate that Δ^9^-THC may facilitate oxycodone-induced antinociception without augmenting certain unwanted features of opioid intake (eg, dependence and circadian rhythm alterations). However, Δ^9^-THC also recruited the rewarding properties of oxycodone at therapeutically relevant doses. These reward-related findings indicate that Δ^9^-THC and oxycodone combinations may carry increased abuse liability, a primary consideration for their therapeutic evaluation.

**Significance Statement:**

In mice, Δ^9^-tetrahydrocannabinol (Δ^9^-THC) produced a potentiation-like effect on oxycodone-induced antinociception and delayed antinociceptive tolerance without exacerbating opioid dependence or circadian disruption, but Δ^9^-THC also produced conditioned place preference and potentiated nucleus accumbens dopamine release at subeffective oxycodone doses. These findings indicate that Δ^9^-THC may enhance oxycodone-induced antinociception, but also that Δ^9^-THC recruits the rewarding properties of oxycodone at doses that are otherwise subeffective. The increased abuse liability of such combinations is a primary consideration for their therapeutic evaluation.

## 1 Introduction

Oxycodone remains one of the most prescribed analgesics for moderate-to-severe pain.^1^ Similar to other opioids, oxycodone exerts its analgesic actions primarily through the *μ*-opioid receptor.^2^^,^^3^ Long-term use of *μ*-opioid agonists can result in tolerance, sleep–wake disturbances, physical dependence, and opioid use disorder.^4^ Alternative therapeutic interventions for pain are therefore urgently needed.

Cannabis and its derivative Δ^9^-tetrahydrocannabinol (Δ^9^-THC) have been suggested as therapeutic alternatives. Cannabis and cannabis-derived products are now available for medicinal use in most US states^5^ and ∼30% of adults with chronic pain in states with legalized medicinal cannabis report using cannabis for pain.^6^ In preclinical models, Δ^9^-THC produces antinociception in several pain models,^7^ acting primarily through cannabinoid type-1 (CB1) and type-2 receptors.^8^ However, similar to *μ*-opioid agonists, prolonged administration of Δ^9^-THC and other CB1 agonists produces antinociceptive tolerance and dependence, limiting the therapeutic window of CB1 agonists. Better approaches to maximize the therapeutic potential of cannabinoid-based therapies are needed.

There is considerable interest in combination cannabinoid–opioid therapies for pain. CB1 and *μ*-opioid receptors are coexpressed in areas throughout the pain neuraxis where they are poised to interact in the context of antinociception^9^ and antinociceptive tolerance.^10^ A growing body of preclinical evidence demonstrates that coadministration of CB1 and *μ*-agonists produces potentiated antinociception across pain models^11^, ^12^, ^13^, ^14^, ^15^; a recent meta-analysis reported a 3.5-fold reduction in the morphine dose required to produce antinociception when combined with Δ^9^-THC in rodent studies.^15^ Antinociceptive facilitation is also observed in nonhuman primates^13^^,^^16^ and in some human studies,^17^ although a recent review concluded that cannabinoid–opioid combinations are mixed in their analgesic effectiveness in humans and may produce unwanted interactions on drug-liking.^18^

Cannabis legalization may also impact opioid misuse and overdose. Some studies report fewer opioid overdose deaths in states with legal cannabis,^19^ whereas others suggest cannabis access may increase risk of opioid misuse and use disorder.^20^ CB1 and *μ*-opioid receptors are coexpressed in the ventral tegmental area and nucleus accumbens (NAc), regions critical for the rewarding properties of reinforcing drugs.^21^ In rodents, Δ^9^-THC has been reported to both facilitate^22^ and reduce^23^ heroin self-administration, and injected or vaporized Δ^9^-THC reduces oxycodone self-administration.^24^ In nonhuman primates, Δ^9^-THC decreases heroin responsivity but has no effect on fentanyl reward,^25^ suggesting ligand-specific interactions. In humans, smoked cannabis facilitates oxycodone drug-liking,^17^ whereas oral dronabinol reduces oxycodone drug-liking,^26^ a difference likely driven by route and dose. Together, these data indicate that Δ^9^-THC and oxycodone reward interactions are complex and warrant evaluation alongside antinociception.

CB1 and *μ*-opioid receptors are also coexpressed in regions relevant to drug withdrawal.^27^ CB1 agonists, allosteric modulators, and endocannabinoid degradation inhibitors reduce measures of *μ*-opioid agonist dependence and withdrawal.^28^ In humans, dronabinol suppresses opioid withdrawal signs in opioid-dependent individuals,^29^ although it can produce unwanted effects such as tachycardia at withdrawal-effective doses.^26^ Preclinical evaluation of oxycodone and Δ^9^-THC interactions in the context of withdrawal is therefore likely to be translationally informative.

Prior literature indicates that cannabinoids and opioids interact functionally on measures of antinociception, withdrawal, and reward, with ligand, dose, and regimen all influencing the outcome. We therefore evaluated the coadministration of oxycodone and Δ^9^-THC in mice across clinically relevant behaviors: antinociception, reward, and *μ*-opioid agonist withdrawal.

## 2 Materials and methods

### 2.1 Subjects

All experiments used C57BL/6J male and female mice bred in-house or purchased from Jackson Laboratories. Mice were 8–10 weeks of age at the start of experiments. Mice were group-housed 3–5 per cage in all experiments except for the hotplate combination dosing experiments, in which mice were individually housed. Individual housing was required in those experiments because the same cohorts carried home cage circadian activity monitoring, which cannot resolve individual subjects when multiple mice are present in a cage (see section Combination Dosing Experimental Time Course). Mice were maintained in a temperature-controlled facility with a 12-hour light/dark cycle (lights on from 6 AM to 6 PM) and given ad libitum food and water. All experimental procedures were approved by the Washington University Institutional Animal Care and Use Committee and followed the guidelines of the International Association for the Study of Pain. Male and female cohorts were always tested separately. Behavioral testing occurred between 8 am and 6 pm (2:00 and 12:00 ZT). All animal experiments and reporting comply with the Animal Research: Reporting of In Vivo Experiments (ARRIVE) 2.0 guidelines.

### 2.2 Drugs and chemicals

Δ^9^-THC (National Institute on Drug Abuse Drug Supply Program) was provided in ethanol (200 mg/mL), and dissolved in a vehicle of 4.75% ethanol (200 proof; Decon Labs), 5% Kolliphor EL (Sigma-Aldrich), and 90% sterile saline (0.9% NaCl for injection USP; Hospira) and administered at a volume of 5 mL/kg. For this vehicle, a stock of Kolliphor and saline was separately mixed and then added to the corresponding amount of ethanol to yield the final injectable vehicle. Oxycodone (Sigma-Aldrich) was dissolved in sterile saline and administered at a volume of 5 mL/kg. For experiments that involved combinations of Δ^9^-THC and oxycodone, both compounds were dissolved in the same vehicle (4.75% ethanol, 5% Kolliphor EL, and 90% sterile saline). Equal parts of each solution were combined to generate a cocktail solution of Δ^9^-THC and oxycodone, which was injected at a volume of 5 mL/kg.

### 2.3 Hotplate test of antinociception

The hotplate test was used to evaluate antinociception as both oxycodone and Δ^9^-THC have been demonstrated to be antinociceptive in this assay.^30^ In brief, mice were placed on a hotplate (Model PE34 Series 8, IITC Life Science Inc) set to a temperature of 55°C. The latency to the first nocifensive response (hind paw lick or hind paw flick) was recorded. Experimenters (R.A.S. and J.G.W.) were blind to treatment condition during testing and scoring. A maximal cutoff latency of 15 seconds was used to prevent tissue damage.

### 2.4 Measuring oxycodone and Δ^9^-THC acute antinociception and tolerance

Cages of group-housed mice were assigned to receive oxycodone or Δ^9^-THC. Subjects in each cage were randomly assigned to receive a dose of oxycodone, Δ^9^-THC, or their respective vehicle. Oxycodone and Δ^9^-THC were never administered to mice within the same cage; only the doses varied between subjects. Oxycodone or Δ^9^-THC was administered at doses of 1, 3, 10, or 30 mg/kg s.c. twice daily during the light cycle at approximately 8:30 am and 5:30 pm for 5 consecutive days. Hotplate thresholds were measured 30, 60, 90, 180, 360, and 1440 minutes after the first daily injection on days 1, 3, and 5 of repeated dosing. Dose-response curves and ED_50_ values were generated from the 30 minutes timepoint on day 1 of dosing. The 30 minutes timepoint was selected because it corresponded to the peak antinociceptive effect of both compounds, and day 1 was selected because it precedes the onset of antinociceptive tolerance; curves generated at later timepoints or on subsequent dosing days would reflect a combination of potency and tolerance rather than potency alone.

For combination experiments, mice were randomized into groups that received either oxycodone (3 mg/kg s.c.), Δ^9^-THC (3 mg/kg s.c.), combination of oxycodone (3 mg/kg s.c.) + Δ^9^-THC (3 mg/kg s.c.), or vehicle. Drug treatment and hotplate testing were conducted as described above. Group sizes consisted of 5 subjects per group.

### 2.5 Evaluation of μ-opioid receptor agonist dependence

Mice were treated with a final dose of the assigned treatment condition followed by naloxone (3 mg/kg s.c.) 1 hour later. Immediately following naloxone injection, mice were placed into a plexiglass observation chamber and were recorded with a Sony Handycam set at 30 frames per second with 1080p resolution. DeepLabCut (DLC)^31^^,^^32^ was used to conduct markerless pose estimation as described in Goodwin et al.^34^ The network was built from 20 frames extracted from 30 videos using *k*-means clustering and labeled with the following 9 body parts: left ear, right ear, left forepaw, right forepaw, left hind paw, right hind paw, snout, tail base, and back. The training fraction was set to 0.95, and the resnet_50 network was trained for 800,000 iterations. A train error of 1.82 and test error of 6.67 were achieved with a cutoff value of *P* = .6. From this point, the trained network was locked and used to analyze subsequent videos.

Simple behavioral analysis (SimBA)^34^ was used to process DLC data as previously described.^33^ Videos were cropped for occasions that contained frames of jumping and other behaviors, typically around 1 minute long. Frames were labeled for jumping behavior (any instance in which all 4 paws left the platform). Random forest classification was then used to generate a behavioral classifier for jumping behavior with the probability set to 0.9 and behavior bout 35 milliseconds. This classifier was then applied to subsequent videos to generate the total number of jumping instances.

### 2.6 Home cage activity monitoring using passive infrared

To evaluate circadian activity, mice were singly housed, and a passive infrared (PIR)–based activity sensor (Pallidus MR1) was placed in the home cage to detect changes in activity as reported previously.^33^ Briefly, the PIR sensor tested for activity in the home cage 5 times per second, and data were aggregated as the percentage of time that the mouse was active in each 1 minutes bin. To evaluate the acute effect of a single drug injection on activity, the percentage of time active was analyzed as 5 minutes bins (Supplemental Fig. 1). To evaluate general effects on circadian rhythm throughout each experimental phase, activity percentage was analyzed in 1-hour bins throughout the entire light:dark cycle (see Supplemental Fig. 2 for collapsed averages throughout each phase of the experiment). Data were processed on the MR1 and transmitted wirelessly a cloud-based server for storage and visualization. Mice were monitored continuously for 2 days prior to the chronic dosing period.

### 2.7 Combination dosing experimental time course

Mice were individually housed for combination dosing experiments to allow for evaluation of circadian activity as detailed above. Mice were individually housed on day 0 and allowed to habituate to cages with PIR sensors for 1 week, which was noted as the “baseline” stage for evaluation of circadian activity. Mice were then randomized to treatment groups to receive vehicle, oxycodone (3 mg/kg s.c.), Δ^9^-THC (3 mg/kg s.c.), or a combination of oxycodone (3 mg/kg s.c.) and Δ^9^-THC (3 mg/kg s.c.) twice daily during the light cycle as detailed above for 5 days. On day 6 (ie, “withdrawal” day), mice were evaluated for *μ*-opioid receptor agonist dependence as detailed above. Mice were then left undisturbed in their cages for an additional 2 days, noted as the “recovery” stage.

### 2.8 Conditioned place preference and locomotor sensitization

Conditioned place preference (CPP) was performed as previously described.^35^ The apparatus consisted of 2 chambers with distinct visual cues (vertical or horizontal black and white stripes) and 1 center chamber. Each chamber was filled with bedding, and the chambers were wiped down with water, and any soiled bedding was removed between sessions.

Mice were group-housed, and randomly assigned to receive 1 of 7 treatment conditions (vehicle, Δ^9^-THC [3 mg/kg s.c.], oxycodone [1 or 3 mg/kg s.c.], oxycodone [1 or 3 mg/kg s.c.] + Δ^9^-THC [3 mg/kg s.c.]). Multiple treatment conditions were present per cage.

#### 2.8.1 Habituation and baseline chamber preference

On days 1 and 2, mice were allowed access to all 3 chambers for 30 minutes, and the baseline values for time spent in each chamber were derived from day 2. Vehicle and drug chamber pairings for each mouse were then randomly assigned.

#### 2.8.2 Pairing

Days 3–5 served as chamber pairing days. On these days, mice were first injected with vehicle in the ante meridiem (AM) and confined to the designated chamber for 30 minutes. At least 4 hours later, mice were injected with the assigned drug condition and were confined to the opposite chamber for 30 minutes.

#### 2.8.3 Post-test

On day 6, mice were again allowed free access to all 3 chambers for 30 minutes.

Video was recorded using a camera (ZR90; Canon) placed at the top of each chamber at rate of 25 frames per second using Bonsai open-source software.^36^ Videos were then analyzed using EthoVision 14 (Noldus Information Technology). Ethovision was used to calculate the amount of time spent in each chamber and the amount of distance traveled during conditioned sessions.

### 2.9 Stereotaxic surgery and viral injection

For NAc dopamine recordings, mice were anesthetized with isoflurane (1.5%–2%) and head-fixed in a stereotaxic frame. AAV-hSyn-GRAB-DA3m^37^ was injected unilaterally into the NAc (coordinates relative to bregma: anterior-posterior +1.6 mm, medial-lateral ±0.9 mm, dorsal-ventral −4.5 mm) at a volume of 300 nL. A fiber optic cannula (200-*μ*m core diameter, 0.37 numerical aperture, 6-mm length; RWD Life Science) was lowered to a position 100 *μ*m above the injection site and secured with Metabond and dental cement. Mice recovered for ≥3 weeks before recordings to allow viral expression.

### 2.10 Fiber photometry data acquisition

Fluorescence signals were acquired using an RWD tricolor fiber photometry system and accompanying hardware (RWD Life Science) with simultaneous excitation at 470 nm (dopamine-dependent GPCR-activation–based dopamine [GRAB-DA] sensor channel) and 410 nm (motion and bleaching reference channel), and data collected via the accompanying RWD acquisition software at approximately 15 Hz per channel. Each recording session consisted of a 10 minutes preinjection baseline followed by subcutaneous administration of the assigned treatment (vehicle, oxycodone 1 mg/kg, oxycodone 3 mg/kg, Δ^9^-THC 3 mg/kg, or the combination of oxycodone 1 mg/kg + Δ^9^-THC 3 mg/kg) and a 60 minutes postinjection recording window. Injection time was marked by a transistor-transistor logic (TTL) pulse logged in the rig event stream. Conditions were tested in the same animals using a within-subjects, randomized crossover design with 2–3 days between sessions to allow drug washout. Recordings were obtained from 5 mice (2 male and 3 female); all 5 conditions were tested in every subject (complete within-subjects crossover, *n* = 5 per condition).

### 2.11 Photometry signal is preprocessing

Raw fluorescence traces were preprocessed in Python (scipy.optimize.curve_fit and scipy.stats.linregress). For each channel (470 nm signal and 410 nm isosbestic reference), a double-exponential decay (f(t) = c + a_slow·exp[−t/τ_slow] + a_fast·exp[−t/τ_fast]) was fit and subtracted to correct photobleaching. A linear regression between the debleached 410 and 470 nm channels yielded a slope and intercept that were used to derive a fitted reference trace; this fitted reference was subtracted from the debleached 470 nm signal to remove residual motion artifact.^38^ The resulting motion-corrected fluorescence trace was resampled to 5 Hz via linear interpolation.

### 2.12 Photometry analysis

For each session, the motion-corrected fluorescence trace was sliced from −10 to +60 minutes relative to the injection TTL and Z-scored against the mean and SD of the 10 minutes preinjection baseline. Z-scored traces were binned into 3 minutes averages for both the panel-level group comparisons and the per-animal heatmap with bin centers used as the time axis in both cases. Postinjection area under the curve (AUC) was computed as the integral of the binned Z-score time course (z·minute) over six 10 minutes subwindows (0–10, 10–20, 20–30, 30–40, 40–50, and 50–60 minutes). For visualization, per-animal 3 minutes binned Z-score traces were assembled into a heatmap with rows representing individual animals (sorted by descending postinjection AUC within each treatment condition) and columns representing 3-minute bins from −10 to +60 minutes relative to injection.

### 2.13 Statistical analysis

The experimenters (R.A.S. and J.G.W.) were blind to the behavioral treatment condition. All data were analyzed using GraphPad Prism 9.0 and Excel. Raw data for hotplate analysis and ED_50_ calculations were converted to percent maximum possible effect using the following formula: percent maximum possible effect = [(Experimental Latency − Baseline Latency) / (15 − Baseline Latency)] × 100. ED_50_ values were generated using nonlinear regression analysis in GraphPad 9.0. ED_50_ values were compared between sexes for each compound using the extra sum-of-squares *F* test, which tests whether a single ED_50_ fits both datasets better than separate values. Five minutes bins of activity counts were used to demonstrate acute changes in activity (Supplemental Fig. 1). For circadian experiments, activity counts were comprised of 1 hour bins for the entirety of the light:dark cycle. Twenty-four-hour data was transformed into AUC for each light:dark period (12 hours). Total percentage of time active during the light phase was calculated by dividing the AUC of the light activity during the 12-hour “light-on period” by the total AUC for that day. For CPP experiments, raw values for drug and vehicle chambers were compared as we have done previously.^14^ We also calculated a preference score (postchamber values − prechamber values) (data not shown). Results between these 2 statistical measures were congruent as such, only the raw values are plotted herein. Behavioral data were analyzed via an ANOVA (two-way or one-way) followed by Tukey’s or Sidak’s post hoc tests. Relevant statistically significant comparisons are reported in the text and represented in the figure legends. For photometry experiments, Z-score time courses were compared between conditions using per-bin paired *t* tests. Subwindow AUC was analyzed by repeated-measures one-way ANOVA followed by paired post hoc *t* tests (Holm-Bonferroni corrected). The formal test of potentiation was a repeated-measures 2 × 2 factorial ANOVA on the vehicle/oxycodone 1 mg/kg / Δ^9^-THC 3 mg/kg/oxycodone (1 mg/kg) + Δ^9^-THC (3 mg/kg) dose-addition design,^39^ with oxycodone (0 vs 1 mg/kg) and Δ^9^-THC (0 vs 3 mg/kg) as factors and their interaction term as the index of nonadditive potentiation. The oxycodone 3 mg/kg condition was included as a positive-control reference and excluded from the factorial. Planned pairwise contrasts (oxycodone [1 mg/kg] + Δ^9^-THC [3 mg/kg] vs oxycodone 1 mg/kg, vs vehicle, vs oxycodone 3 mg/kg) used paired *t* tests with Holm-Bonferroni correction. Photometry analyses were performed in Python using statsmodels, Pingouin, and SciPy. Detailed statistics are reported in tables. Detailed statistical results for each figure are provided in the accompanying Supplemental Tables 1–8.

## 3 Results

### 3.1 Oxycodone and Δ^9^-THC produce dose-dependent antinociception in the hotplate test

To evaluate antinociceptive potency and tolerance, separate groups of male and female mice were administered oxycodone (1, 3, 10, and 30 mg/kg s.c.), Δ^9^-THC (1, 3, 10, and 30 mg/kg s.c.), or their respective vehicles twice daily for a period of 5 days (see Fig. 1A and Fig. 2A for timeline). Dose-response relationships were derived from the 30 minutes postinjection timepoint on day 1 of dosing. Oxycodone produced dose-dependent antinociception in both males (ED_50_ = 2.81 [1.90–4.39]; Fig. 1B) and females (ED_50_ = 5.17 [3.83–7.00]; Fig. 1C). The ED_50_ values were higher in females, and ED_50_ differed significantly between sexes (extra sum-of-squares *F* test, *F*_1,53_ = 4.65, *P* = .0356). Similarly, Δ^9^-THC produced dose-dependent antinociception in both males (ED_50_ = 12.53 [8.24–19.28]; Fig. 2B) and females (ED_50_ = 19.20 [11.90–31.81]; Fig. 2C). Again, ED_50_ values in females were larger; ED_50_ did not differ significantly between sexes (extra sum-of-squares *F* test, *F*_1,58_ = 0.90; *P* = .3475).

**Fig. 1:**
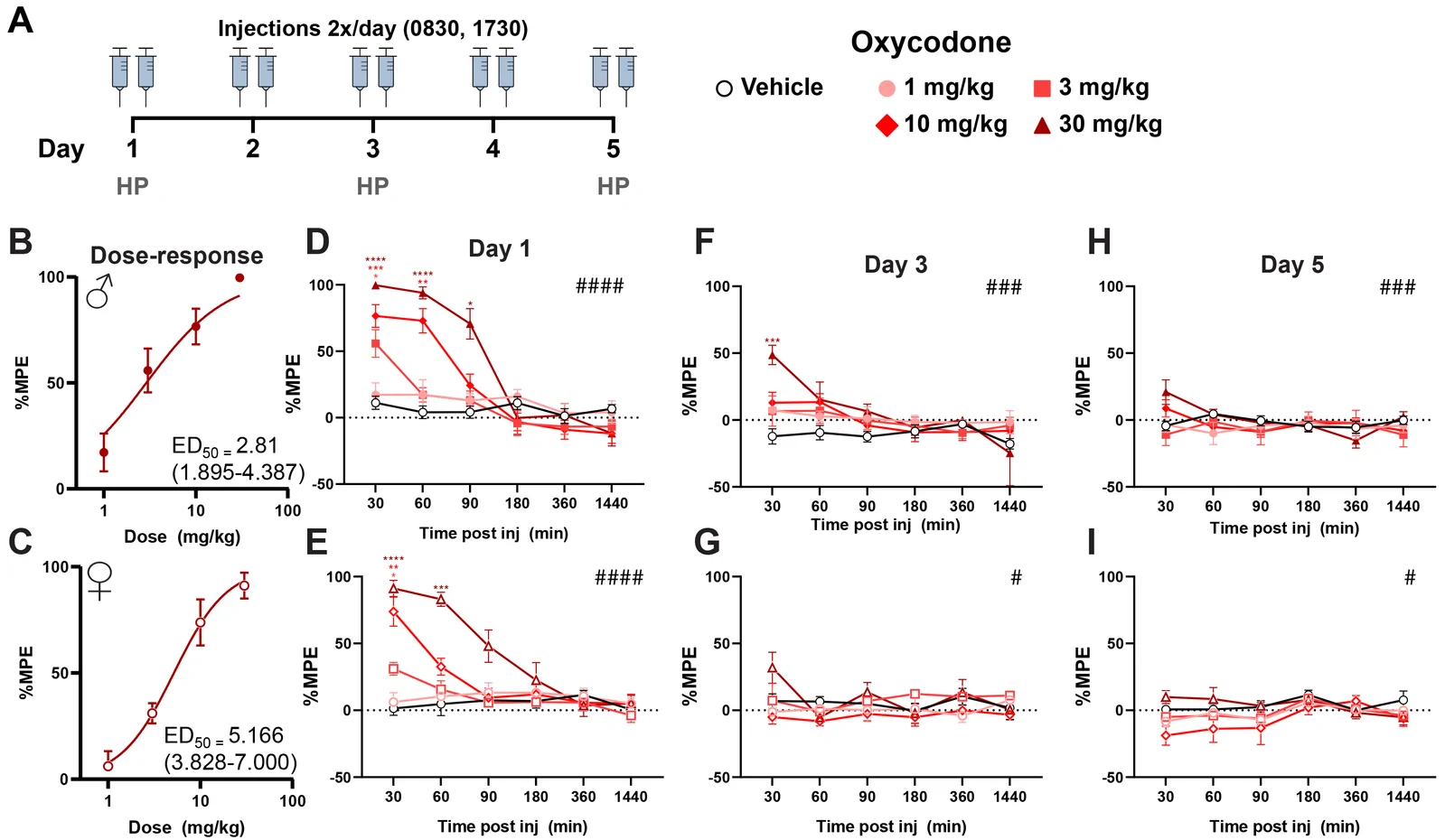
Oxycodone produces dose-dependent antinociception in the hotplate test. Oxycodone (1, 3, 10, and 30 mg/kg s.c.) was administered twice daily over a period of 5 days (A). Oxycodone produced dose-dependent antinociception in males (B) and females (C) at the 30 minutes timepoint on day 1 of dosing, expressed as percent maximum possible effect (%MPE). Time course of antinociception on day 1 is shown for males (D) and females (E). Tolerance developed by day 3 to most doses (males: F, females: G) and to all doses by day 5 (males: H, females: I). Data are represented as mean ± SEM. *n* = 6–7 per group. Colored asterisks denote Tukey’s post hoc comparisons against vehicle at the indicated timepoint for the dose of the corresponding color (∗*P* < .05, ∗∗*P* < .01, ∗∗∗*P* < .001, and ∗∗∗∗ *P* < .0001). Black symbols denote the treatment × time interaction for that panel (^#^*P* < .05, ^###^*P* < .001, and ^####^*P* < .0001), two-way repeated-measures ANOVA. Complete pairwise comparisons for every timepoint at which any comparison reached significance are reported in Supplemental Table 1.

**Fig. 2:**
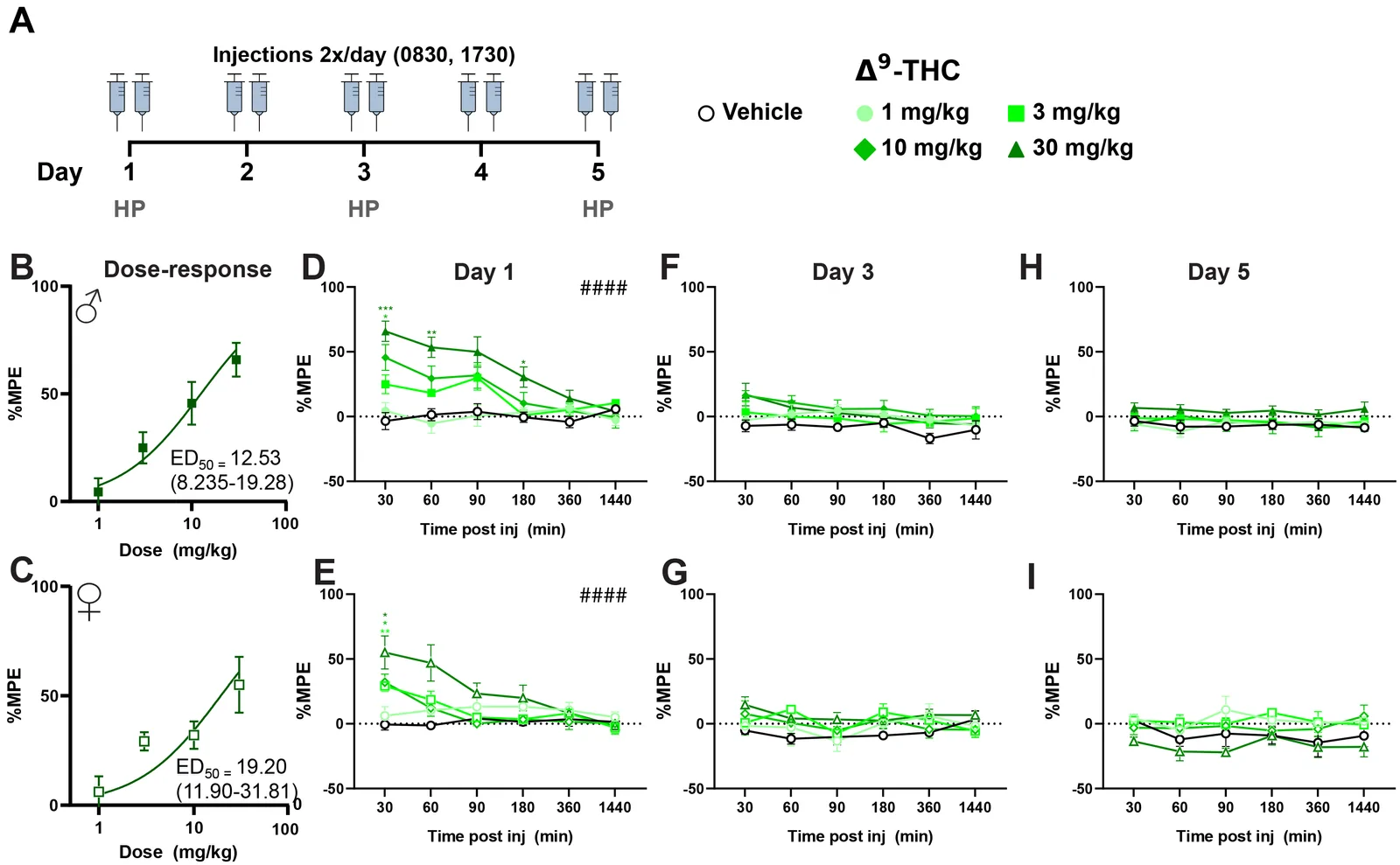
Δ^9^-THC produces dose-dependent antinociception in the hotplate test. Δ^9^-THC (1, 3, 10, and 30 mg/kg s.c.) was administered twice daily over a period of 5 days (A). Δ^9^-THC produced dose-dependent antinociception in males (B) and females (C) at the 30 minutes timepoint on day 1 of dosing, expressed as percent maximum possible effect (%MPE). Time course of antinociception on day 1 is shown for males (D) and females (E). Tolerance developed by day 3 (males: F, females: G) and remained by day 5 (males: H, females: I) in both sexes. Data are represented as mean ± SEM. *n* = 6–7 per group. Colored asterisks denote Tukey’s post hoc comparisons against vehicle at the indicated timepoint for the dose of the corresponding color (∗*P* < .05, ∗∗ *P* < .01, and ∗∗∗ *P* < .001). Black symbols denote the treatment × time interaction for that panel (^####^*P* < .0001), two-way repeated-measures ANOVA. Complete pairwise comparisons for every timepoint at which any comparison reached significance are reported in Supplemental Table 2.

To determine the time course of oxycodone and Δ^9^-THC-induced antinociception, hotplate latencies were measured at 30, 60, 90, 180, 360, and 1440 minutes postinjection. At the highest dose tested (30 mg/kg s.c.), the oxycodone-induced antinociceptive effect lasted until 90 minutes postinjection in males (*P* < .05 vs vehicle two-way ANOVA followed by Tukey’s post hoc test; Fig. 1D; Supplemental Table 1) and 60 minutes postinjection in females (*P* < .01 vs vehicle two-way ANOVA followed by Tukey’s post hoc test; Fig. 1E; Supplemental Table 1). Similar to oxycodone, Δ^9^-THC produced antinociception at 30 mg/kg s.c. dose for up to 60 minutes postinjection in males (*P* < .01 two-way ANOVA followed by Tukey’s post hoc test; Fig. 2D; Supplemental Table 2) but only 30 minutes postinjection for females (*P* < .05 two-way ANOVA followed by Tukey’s post hoc; Fig. 2E; Supplemental Table 2). In males, the 30 mg/kg comparison reached significance again at 180 minutes (*P* = .0494) despite being nonsignificant at 90 minutes; we regard this nonmonotonic result as unlikely to reflect sustained antinociception and do not interpret it further.

By day 3 of dosing, antinociceptive tolerance had developed to all doses of oxycodone except the highest dose tested (30 mg/kg s.c.) in both males (Fig. 1F; Supplemental Table 1) and females (Fig. 1G; Supplemental Table 1). By day 5, all doses were ineffective relative to vehicle (Fig. 1, H and I). Δ^9^-THC-treated mice developed tolerance by day 3 in both males (Fig. 2F) and females (Fig. 2G) that remained on day 5 of dosing (Fig. 2, H and I; Supplemental Table 2).

### 3.2 Δ^9^-THC produces a potentiation-like effect on oxycodone-induced antinociception and delays the development of antinociceptive tolerance

Next, we sought to evaluate the effect of coadministration of Δ^9^-THC and oxycodone on hotplate antinociception and antinociceptive tolerance. To this end, we used the dose-response curves generated in Figs. 1 and 2 to incorporate a dose of Δ^9^-THC that does not produce antinociception (3 mg/kg s.c.), combined with an effective but not maximal antinociceptive dose of oxycodone (3 mg/kg s.c.). Mice received either oxycodone (3 mg/kg s.c.), Δ^9^-THC (3 mg/kg s.c.), a combination of oxycodone (3 mg/kg s.c.) and Δ^9^-THC (3 mg/kg s.c.), or vehicle twice daily for a period of 5 days (see Fig. 3A for timeline). At these doses Δ^9^-THC appeared to potentiate oxycodone-induced antinociception relative to all groups on day 1 and at the 30 minutes timepoint (males: *F*_15,80_ = 6.97, *P* < .01; females: *F*_15,80_ = 5.60, *P* < .01 two-way ANOVA interaction effect; Fig. 3, B and C; Supplemental Table 3). The statistical basis for this potentiation-like effect is the treatment × time interaction term of the two-way ANOVA within this cohort, in which all 4 groups were run concurrently under identical housing conditions. At the 30 minutes timepoint on day 1, the combination differed from vehicle in both sexes (males: *P* = .0002, females: *P* = .0016), whereas Δ^9^-THC (3 mg/kg s.c.) alone did not differ from vehicle in either sex (males: *P* = .9475, females: *P* = .1566), and oxycodone (3 mg/kg s.c.) alone differed from vehicle in males (*P* = .0038) but not in females (*P* = .0938; Tukey’s post hoc; Supplemental Table 3). A potentiation-like effect was also observed on day 3 at the 30 minutes timepoint (males: *F*_15,80_ = 4.19, *P* < .01; females: *F*_15,80_ = 3.29, *P* < .01 two-way ANOVA interaction effect; Fig. 3, D and E; Supplemental Table 3). These 30 minutes magnitudes are summarized across dosing days in Fig. 3H (males) and Fig. 3I (females), where the combination exceeded each single drug administered alone on days 1 and 3 (all combination-vs-single-drug comparisons *P* < .05, Tukey’s post hoc test; Supplemental Table 3), with the sole exception of one comparison in females on day 3. However, by day 5, there were no differences between groups (Fig. 3, F–I).

**Fig. 3:**
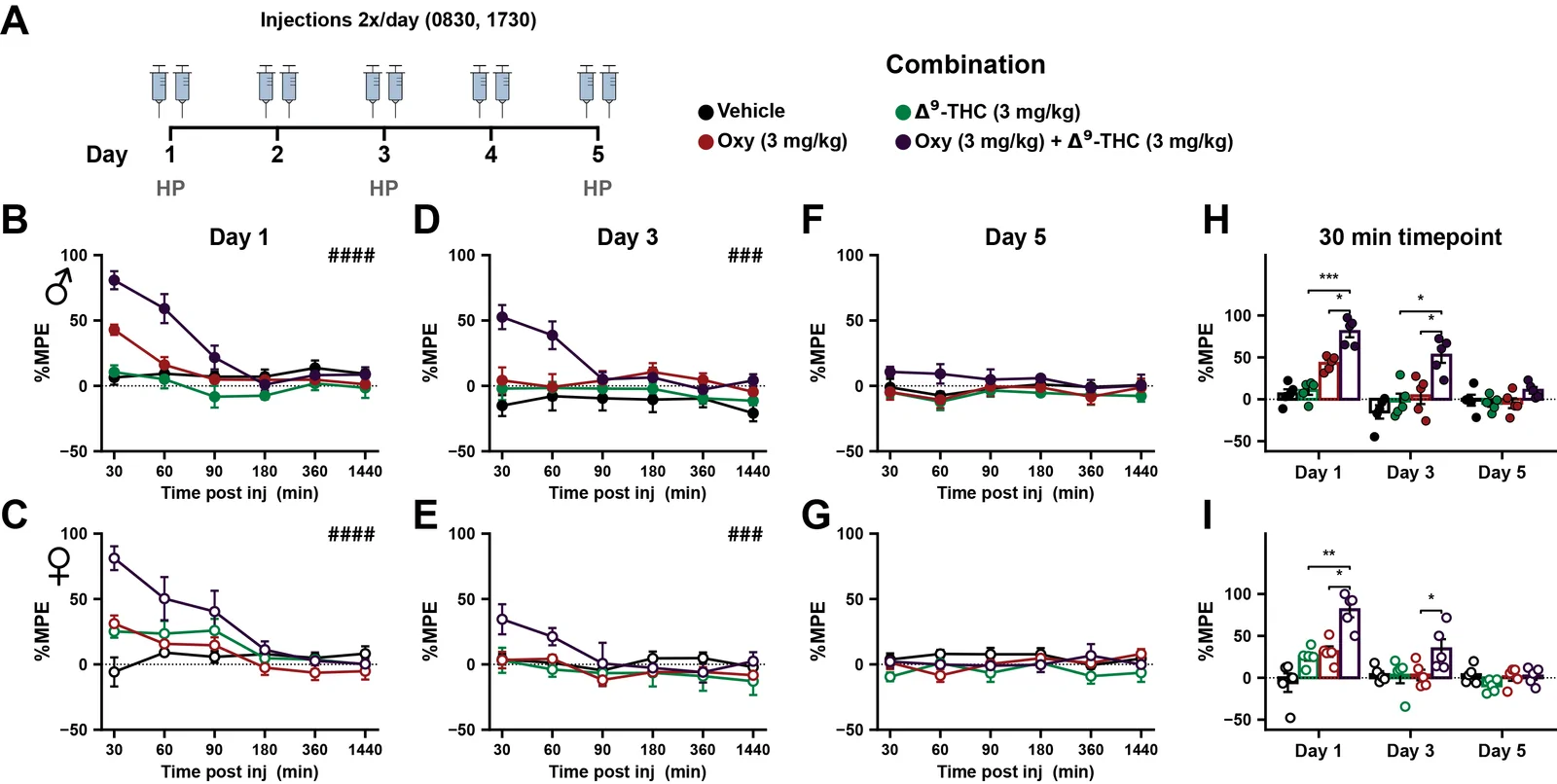
Combination dosing of Δ^9^-THC with oxycodone results in a potentiation-like effect on antinociception and delays antinociceptive tolerance. Schematic of experimental time course (A). Oxycodone (3 mg/kg s.c.) and Δ^9^-THC (3 mg/kg s.c.) produced greater and longer-lasting antinociception when coadministered relative to either administered alone on day 1 of dosing in both males (B) and females (C). On day 3 of dosing, the combination remained efficacious, but tolerance had developed to the other treatment groups in both males (D) and females (E). By day 5, complete tolerance had developed in all groups in both males (F) and females (G). Group means at the 30 minutes (peak) timepoint are summarized for each dosing day in males (H) and females (I); bars are mean ± SEM with individual animals overlaid. On days 1 and 3, the combination produced greater antinociception than either oxycodone or Δ^9^-THC administered alone, an effect absent by day 5; the combination-vs-Δ^9^-THC comparison did not reach significance in females on day 3. Data are represented as mean ± SEM. *n* = 5 per group. ^###^*P* < .001 and ^####^*P* < .0001 denote the treatment × time interaction, two-way ANOVA (B–G); ∗*P* < .05, ∗∗*P* < .01, and ∗∗∗ < .001 denote the combination vs each single drug administered alone, Tukey’s post hoc (H, I).

### 3.3 Repeated dosing with oxycodone produces physical dependence. Combination treatment of Δ^9^-THC and oxycodone reduced oxycodone-induced physical dependence in male but not female mice

We next sought to evaluate whether our repeated dosing regimen would produce *μ*-opioid receptor agonist dependence. Repeated administration of *μ*-opioid agonists produces physical dependence that can be evidenced with administration of naloxone. Mice were administered naloxone (3 mg/kg s.c.) on day 6 of repeated dosing and evaluated for jumping behavior over 30 minutes. Male (*F*_4,24_ = 5.04, *P* < .01, one-way ANOVA; Fig. 4B; Supplemental Table 4) and female (*F*_4,20_ = 4.69, *P* < .01, one-way ANOVA; Fig. 4C; Supplemental Table 4) mice treated with oxycodone exhibited jumping behavior following naloxone treatment, indicative of *μ*-opioid agonist-mediated dependence. Combination treatment with Δ^9^-THC reduced naloxone-precipitated jumping behavior in males (*F*_3,16_ = 7.85, *P* < .01, one-way ANOVA; Fig. 4D; Supplemental Table 4) but not females (*F*_3,16_ = 5.30, *P* < .01, one-way ANOVA; Fig. 4E; Supplemental Table 4).

**Fig. 4:**
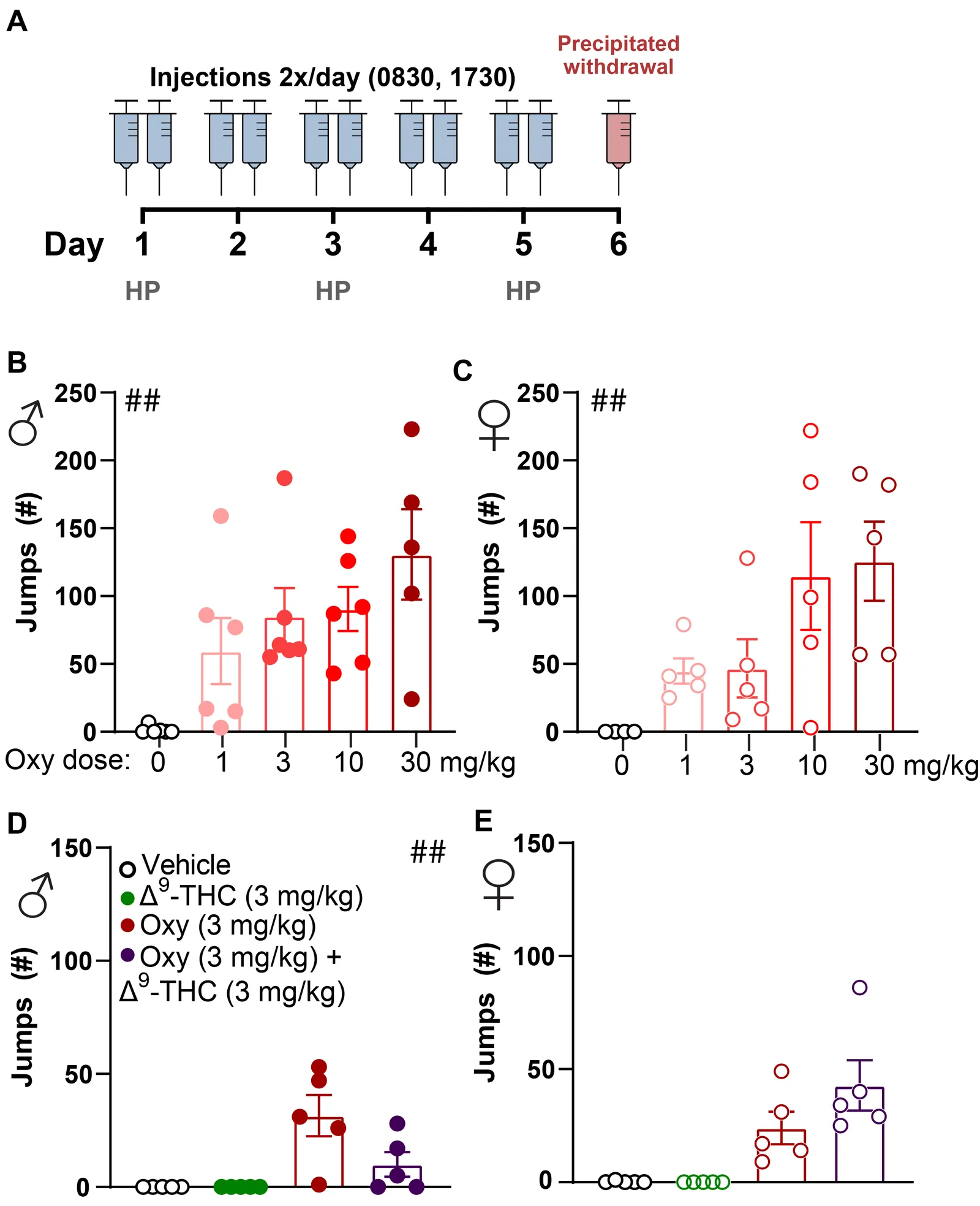
Repeated dosing with oxycodone induces *μ*-opioid receptor agonist dependence; coadministration of oxycodone with Δ^9^-THC reduces *μ*-opioid receptor agonist dependence in males. Schematic of experimental time course (A). Oxycodone produced dependence to a similar degree in both males (B) and females (C) following repeated dosing. Males treated with Δ^9^-THC and oxycodone in combination had a reduced number of jumps (D), whereas the females did not differ from oxycodone alone (E). Data are represented as mean ± SEM. *n* = 5–7 per group. ^##^*P* < .01 denotes the main effect of treatment, one-way ANOVA.

### 3.4 Treatment with oxycodone or a combination of oxycodone and Δ^9^-THC results in alterations of circadian activity

We next sought to evaluate whether our dosing regimen with oxycodone, Δ^9^-THC, or a combination would produce changes in general circadian activity during dosing, withdrawal, and recovery periods (see Fig. 5A for timeline). Mice treated with Δ^9^-THC (3 mg/kg s.c.) did not exhibit alterations in circadian activity relative to vehicle-treated controls in either males (Fig. 5, B and H; Supplemental Table 5) or females (Fig. 5, C and I; Supplemental Table 5). Mice treated with oxycodone (3 mg/kg s.c.) were more active during the dosing period relative to vehicle-treated controls in both sexes (males: interaction *F*_9,48_ = 7.33, *P* < .0001, vehicle vs oxycodone *P* = .0018; females: interaction *F*_9,48_ = 2.36, *P* = .0269, vehicle vs oxycodone *P* = .0275; two-way ANOVA followed by Tukey’s post hoc test; Fig. 5, D, E, H, and I; Supplemental Table 5). Combination treatment of Δ^9^-THC (3 mg/kg s.c.) and oxycodone (3 mg/kg s.c.) resulted in increased activity during the light period in both sexes (males: vehicle vs combination, *P* < .0001; females: *P* = .0151; two-way ANOVA followed by Tukey’s post hoc test; Supplemental Table 5). See Supplemental Fig. 1 for acute activity increase, and Supplemental Fig. 2 for increase throughout the dosing phase. Total activity in the light period was normalized during the 2-day recovery period following withdrawal.

**Fig. 5:**
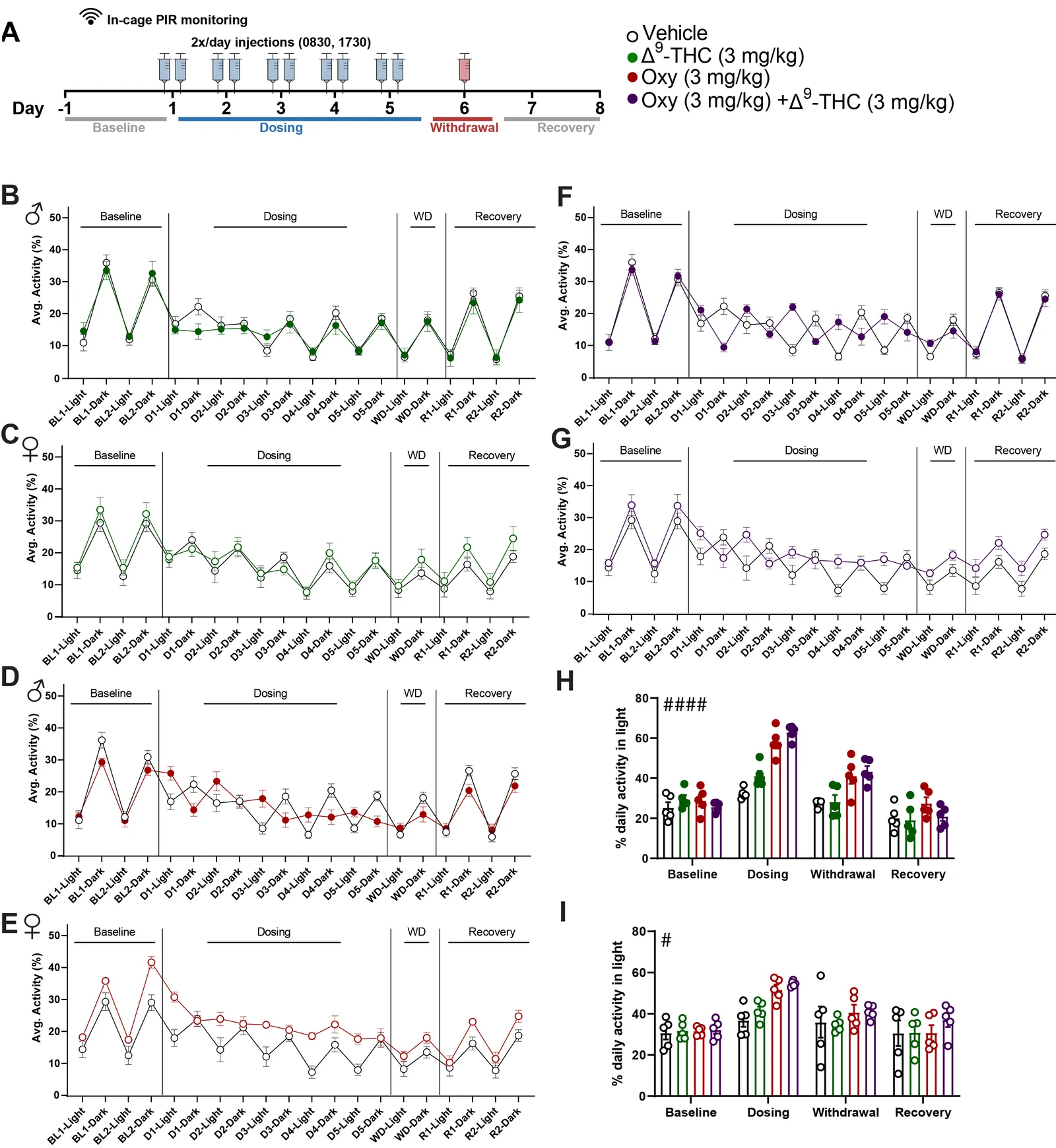
Dosing with oxycodone or a combination of Δ^9^-THC with oxycodone results in disturbances of circadian activity. Schematic of experimental time course (A). Δ^9^-THC had a minimal effect on the general circadian activity pattern relative to vehicle (B, C). Mice treated with oxycodone (D, E) or a combination of oxycodone + Δ^9^-THC (F, G) spent a greater percentage of time active in the light relative to vehicle during the dosing period, which normalized after withdrawal in both males (H) and females (I). Values for percentage of time in light were averaged across each experimental phase and compared in panels (H, I). Data are represented as mean ± SEM. *n* = 5 per group. ^#^*P* < 0.05 and ^####^*P* < .0001 denote the treatment × time interaction, two-way ANOVA.

### 3.5 Δ^9^-THC facilitates oxycodone-induced CPP and does not alter oxycodone-induced locomotor sensitization

Next, we incorporated CPP to evaluate whether Δ^9^-THC would enhance or dampen oxycodone-induced CPP at doses therapeutically relevant for antinociception.

Mice administered vehicle for pairing conditions did not exhibit changes in chamber preference (Fig. 6, A and B). Δ^9^-THC did not produce CPP in either sex (Fig. 6, C and D). Mice administered oxycodone at 1 mg/kg s.c. did not exhibit CPP for the drug-paired chamber (Fig. 6, A and B). In contrast, mice treated with a 3 mg/kg s.c. A dose of oxycodone developed a robust CPP to the drug-paired chamber (male: *P* < .01, female: *P* < .01 vs vehicle-paired chamber two-way ANOVA followed by Bonferroni post hoc test; Fig. 6, G and H; Supplemental Table 6).

**Fig. 6:**
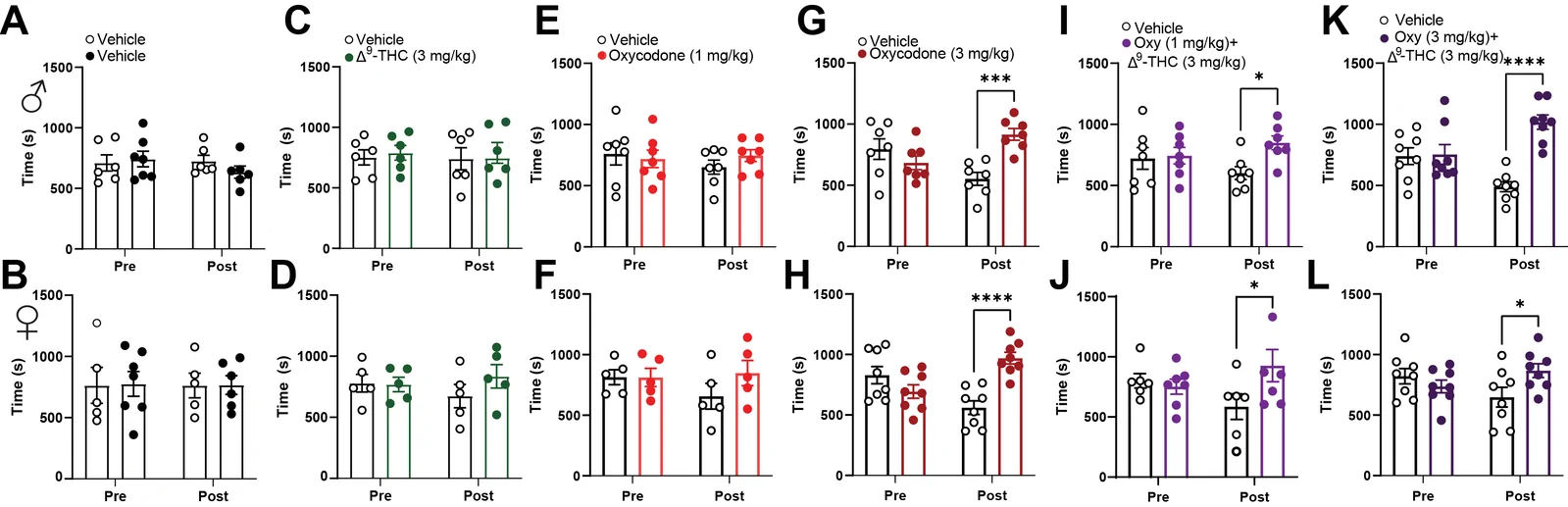
Oxycodone and Δ^9^-THC produce CPP when administered in combination. Administration of vehicle did not result in chamber preference alterations in males (A) or females (B). Administration of Δ^9^-THC did not result in CPP in either males (C) or females (D). Administration of oxycodone (1 mg/kg s.c.) did not produce CPP in males (E) or females (F). By contrast, a 3 mg/kg dose of oxycodone produced CPP in both males (G) and females (H). Doses of Δ^9^-THC (3 mg/kg s.c.) and oxycodone (1 mg/kg s.c.) that did not produce preference on their own produced CPP when coadministered in both males (I) and females (J). Δ^9^-THC (3 mg/kg s.c.) did not alter oxycodone (3 mg/kg s.c.)-induced CPP in either males (K) or females (L). Data are represented as mean ± SEM. *n* = 5–8 per group. ∗∗∗∗*P* < .0001, ∗∗∗*P* < .001, and ∗*P* < .05. 2 × 2 ANOVA followed by Sidak’s post hoc test.

We next evaluated whether doses of oxycodone (1 mg/kg s.c.) and Δ^9^-THC (3 mg/kg s.c.), which did not produce preference alone, would produce preference when coadministered. Mice administered this combination produced CPP in both sexes (male: *P* = .0328, female: *P* = .0234 vs vehicle-paired chamber two-way ANOVA followed by Sidak’s post hoc test; Fig. 6, I and J; Supplemental Table 6). Similarly, combination of a preference-producing oxycodone dose (3 mg/kg s.c.) and Δ^9^-THC (3 mg/kg s.c.) produced CPP (male: *P* < .01, female: *P* < .05 two-way ANOVA followed by Sidak’s post hoc test; Fig. 6, K and L; Supplemental Table 6). These data indicate that Δ^9^-THC does not reduce oxycodone-induced CPP, and subthreshold doses of oxycodone and Δ^9^-THC become preference-producing when combined.

*μ*-Opioid agonists produce an initial hyperlocomotor response that sensitizes following repeated administration.^40^ We tracked locomotion during the CPP pairing days to evaluate this effect across our different groups. Oxycodone produced increased locomotion at 3 mg/kg in both sexes across all pairing days (male: *F*_5,39_ = 43.00, *P* < .01; female: *F*_5,29_ = 12.29, *P* < .01 two-way ANOVA treatment effect; Fig. 7, A and B; Supplemental Table 7). The combination group of oxycodone (3 mg/kg s.c.) with Δ^9^-THC (3 mg/kg s.c.) also produced enhanced locomotion relative to all other groups, aside from the oxycodone (3 mg/kg s.c.) group, in both sexes. Although the low-dose oxycodone (1 mg/kg s.c.) and Δ^9^-THC (3 mg/kg s.c.) combination group produced preference, there was no increase in locomotion or indication of locomotor sensitization in either sex.

**Fig. 7:**
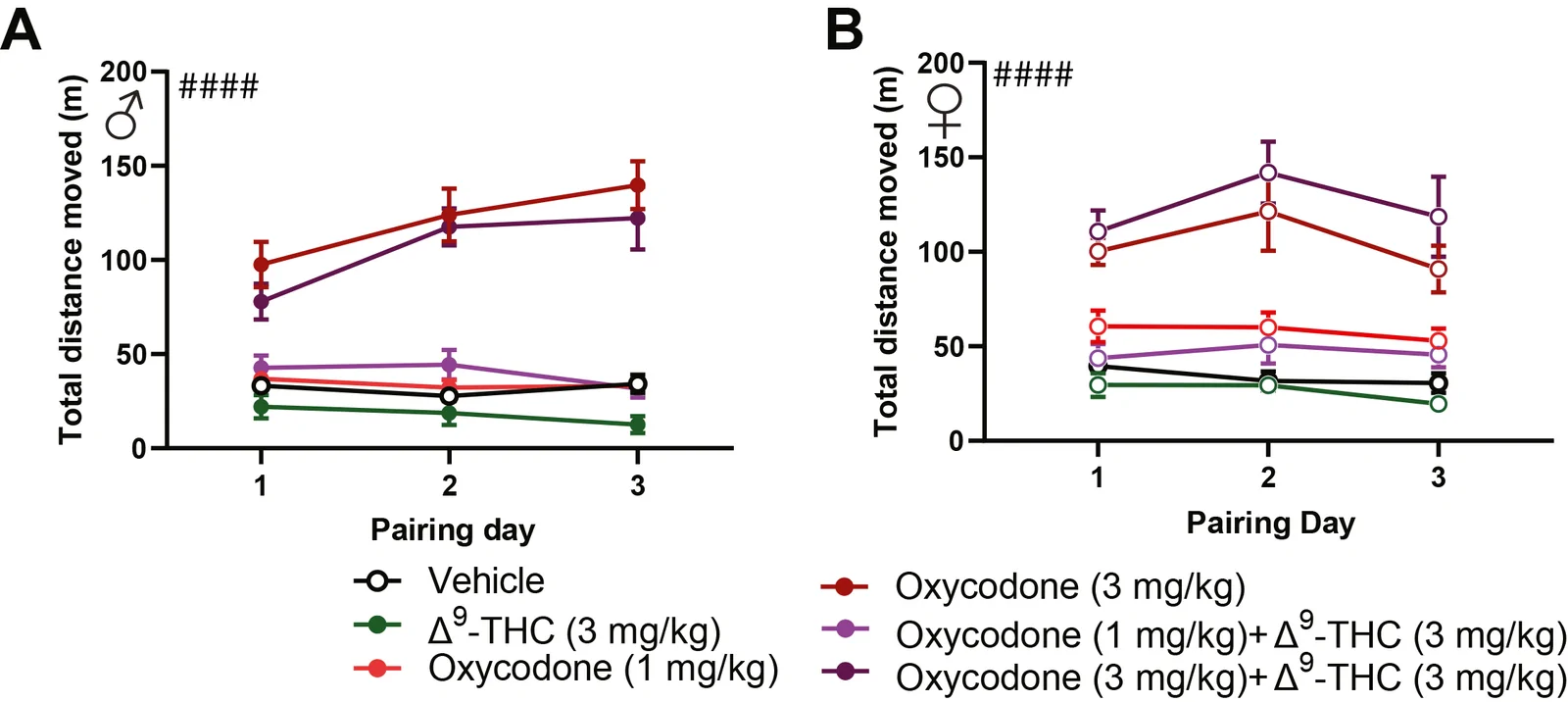
Δ^9^-THC does not alter oxycodone-induced locomotor sensitization. Distance traveled was measured on each pairing day during CPP conditioning. Oxycodone increased distance traveled, whereas Δ^9^-THC had no effect in both males (A) and females (B). Δ^9^-THC also did not alter distance traveled when coadministered with oxycodone. Data are represented as mean ± SEM. *N* = 5–8 per group. ^####^*P* < .0001 denotes the treatment × time interaction, two-way ANOVA.

### 3.6 Δ^9^-THC potentiates oxycodone-evoked NAc dopamine release at subeffective oxycodone doses

To investigate whether the Δ^9^-THC and oxycodone CPP interaction (Fig. 6) was paralleled by changes in NAc dopamine release, we recorded GRAB-DA fluorescence in NAc during single subcutaneous injections of vehicle, oxycodone (1 or 3 mg/kg s.c.), Δ^9^-THC (3 mg/kg s.c.), or the combination of oxycodone (1 mg/kg s.c.) with Δ^9^-THC (3 mg/kg s.c.); the lower oxycodone dose was chosen to mirror the subeffective pairing from the CPP experiment (Fig. 5, I and J). Δ^9^-THC (3 mg/kg s.c.) alone did not differ from vehicle (Fig. 8A; Supplemental Table 8), whereas oxycodone produced a dose-dependent NAc dopamine response (Fig. 8, B and C; Supplemental Table 8). The combination of oxycodone (1 mg/kg s.c.) with Δ^9^-THC (3 mg/kg s.c.) produced a sustained dopamine response that approached the magnitude of oxycodone (3 mg/kg s.c.) alone (Fig. 8D; Supplemental Table 8) and exceeded the oxycodone (1 mg/kg s.c.) response in the sustained midphase of the postinjection window (∼15–30 minutes; Fig. 8E; Supplemental Table 8). A repeated-measures 2 × 2 factorial ANOVA on the vehicle/oxycodone (1 mg/kg s.c.) / Δ^9^-THC (3 mg/kg s.c.) / oxycodone (1 mg/kg s.c.) + Δ^9^-THC (3 mg/kg s.c.) cells confirmed a significant oxycodone × Δ^9^-THC interaction in the 10–20 minutes subwindow (*F*_1,4_ = 20.37, *P* = .0107; Fig. 8F; Supplemental Table 8), indicating potentiation.^39^ Together, these data indicate that Δ^9^-THC potentiates oxycodone-evoked NAc dopamine release at an oxycodone dose that is otherwise insufficient to produce robust dopamine elevation, paralleling the behavioral CPP interaction at the same dose pair.

**Fig. 8:**
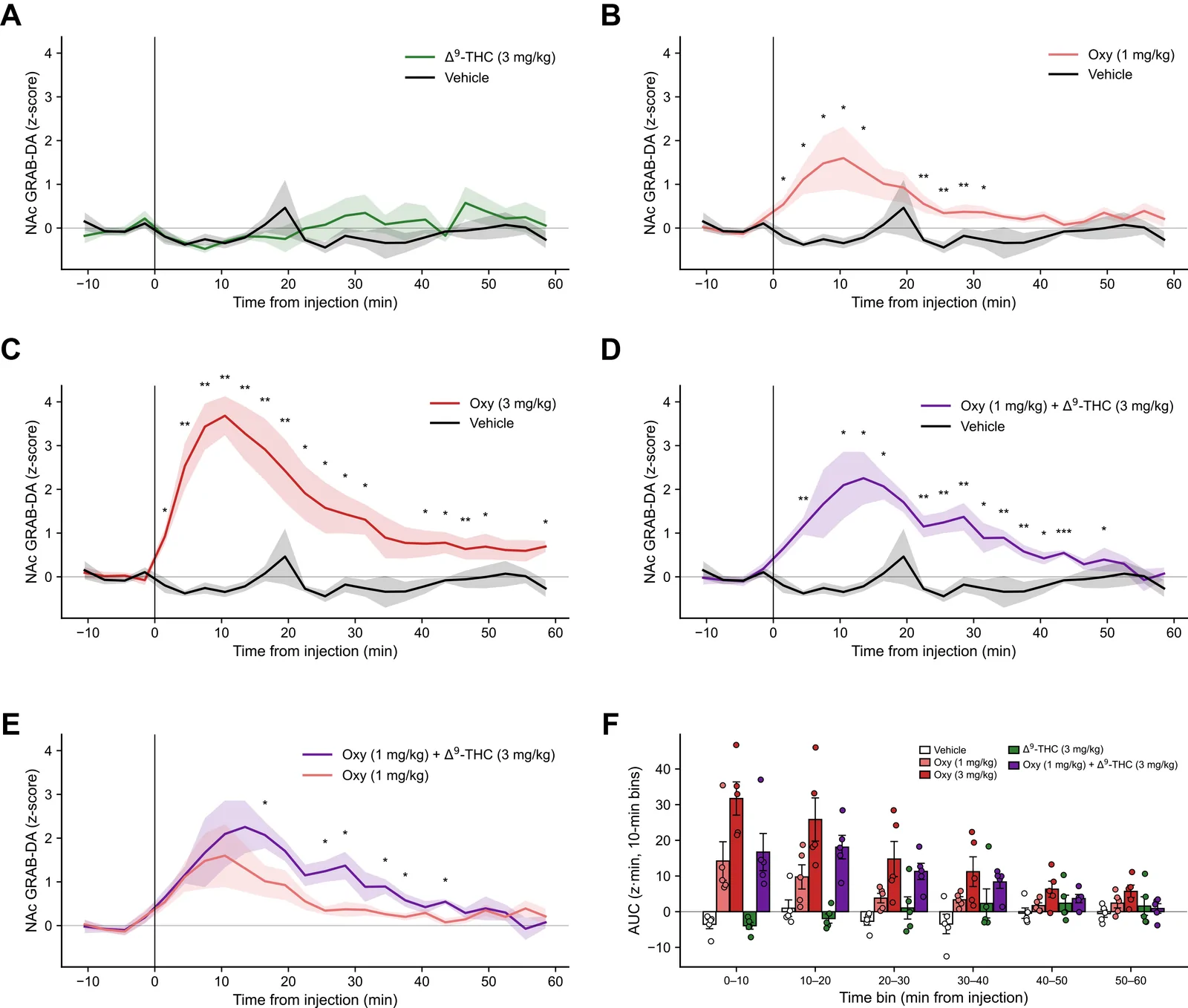
Δ^9^-THC potentiates oxycodone-evoked NAc dopamine release at a subeffective oxycodone dose. Panels (A–E) show pairwise comparisons of NAc GRAB-DA Z-score time courses (3-minute bins; injection at *t* = 0). (A) Δ^9^-THC (3 mg/kg) vs vehicle. (B) Oxycodone (1 mg/kg) vs vehicle. (C) Oxycodone (3 mg/kg) vs vehicle. (D) Oxycodone (1 mg/kg) + Δ^9^-THC (3 mg/kg) vs vehicle. (E) Oxycodone (1 mg/kg) + Δ^9^-THC (3 mg/kg) vs oxycodone (1 mg/kg). (F) Subwindow postinjection AUC in six 10 minutes bins (0–10, 10–20, 20–30, 30–40, 40–50, and 50–60 minutes); bars are mean ± SEM, dots are individual animals. Data are represented as mean ± SEM. Recordings were obtained from 5 mice (2 male, 3 female) in a complete within-subjects crossover (*n* = 5 per condition). ∗*P* < .05, ∗∗*P* < .01, and ∗∗∗*P* < .001 per-bin paired *t* tests.

## 4 Discussion

We demonstrate behaviorally significant interactions of Δ^9^-THC and oxycodone on antinociception and reward. At antinociceptive doses, Δ^9^-THC did not alter oxycodone-induced *μ*-opioid receptor dependence or circadian activity, whereas cotreatment produced CPP at doses that were not preference-producing alone.

Both oxycodone and Δ^9^-THC produced dose-dependent antinociception in the hotplate. Prior reports indicate that females are more sensitive to the antinociceptive effects of Δ^9^-THC,^41^ and have demonstrated differential rates of antinociceptive tolerance.^42^ In our study, Δ^9^-THC produced a higher ED_50_ in females than in males, but the difference was not statistically significant (*F*_1,58_ = 0.90, *P* = .3475). This is consistent with one report of higher female Δ^9^-THC ED_50_ on the tail-flick test in mice,^42^ although it contrasts with reports in rats indicating greater female sensitivity,^41^^,^^43^ likely reflecting a species difference. We observed a significant sex difference in oxycodone-induced antinociception, with lower potency in females (*F*_1,53_ = 4.65, *P* = .0356), consistent with a prior report of lower oxycodone analgesic responsiveness in females at higher doses.^44^ Tolerance developed similarly in both sexes for either compound. Δ^9^-THC antinociceptive tolerance is both stimulus-dependent and dose-dependent; a recent report found that tolerance to the antinociceptive effects of Δ^9^-THC on cold hypersensitivity emerged by day 7 at 3 mg/kg and by day 14 at 1 mg/kg once-daily i.p. dosing, whereas relief of mechanical hypersensitivity was sustained over 14 days.^45^ Because our regimen delivered a far higher dose (up to 60 vs 3 mg/kg/day), the more rapid tolerance we observed is consistent with the dose dependence reported therein. That study, however, assessed mechanical and cold hypersensitivity rather than noxious heat, and used a disease model in which Δ^9^-THC was ∼30-fold more potent than in our naive mice; given the stimulus-dependence of Δ^9^-THC tolerance, direct comparison of dosing regimens across these differences is not warranted. The magnitude of antinociception also declined across dosing days at doses that remained statistically significant, and we cannot exclude a contribution of repeated hotplate testing to this decline. Coadministration of a sub-ED_50_ dose of Δ^9^-THC (3 mg/kg s.c.) with a near-ED_50_ dose of oxycodone (3 mg/kg s.c.) produced a potentiation-like effect on oxycodone-induced antinociception and a delay in tolerance development in both sexes, consistent with prior reports of Δ^9^-THC and opioid combinations in rodents,^11^^,^^12^^,^^15^^,^^46^ nonhuman primates,^47^ and humans.^17^ Two ongoing clinical studies are evaluating Δ^9^-THC and oxycodone interactions in an antinociceptive context, in individuals with fibromyalgia^48^ and in pain-free individuals using the cold pressor test (NCT03679949); whether a significant antinociceptive interaction emerges in those trials will be informative.

Prior reports demonstrate that direct CB1 agonists, allosteric modulators, and endocannabinoid degradation inhibitors reduce somatic and autonomic withdrawal in opioid-dependent animals^27^; cotreatment of Δ^9^-THC with fentanyl also does not alter opioid withdrawal signs in nonhuman primates.^47^ These lines of evidence suggest that Δ^9^-THC would not facilitate, and might reduce, oxycodone withdrawal. Consistent with prior reports,^49^^,^^50^ naloxone elicited jumping in mice treated repeatedly with oxycodone, including at the lowest dose (1 mg/kg s.c.), indicating doses without antinociceptive efficacy are still sufficient to produce dependence. In males, coadministration of Δ^9^-THC reduced oxycodone-elicited jumping; this effect was not observed in females. Δ^9^-THC did not alter locomotor behavior at this dose, so locomotor confounds are unlikely. Together, these results suggest that Δ^9^-THC does not exacerbate *μ*-opioid receptor physical dependence at antinociceptive-facilitating doses and may reduce opioid withdrawal signs.

Alterations in sleep and circadian rhythm occur following repeated intake and withdrawal from opioids and cannabis.^51^ To our knowledge, our study is the first to evaluate the oxycodone–Δ^9^-THC combination on such metrics. Twice-daily oxycodone (3 mg/kg s.c.) markedly altered circadian activity in both sexes, paralleling our prior report on voluntary oral oxycodone consumption,^34^ and normalized following withdrawal. Longer-lasting postwithdrawal effects reported after morphine^52^ or fentanyl^51^ likely reflect their different opioids, regimens, and the absence of precipitated withdrawal in those studies. Δ^9^-THC alone did not affect circadian activity in any phase—differing from reports of sleep changes at higher Δ^9^-THC doses,^53^ likely reflecting endpoint (electroencephalogram vs cage activity), dose, or ligand—and did not alter oxycodone’s circadian impact.

Prior reports demonstrate cannabinoid–opioid interactions on reward in rodents,^54^^,^^55^ nonhuman primates,^16^ and humans.^17^ In our study, oxycodone produced dose-dependent CPP in both sexes at 3 mg/kg, consistent with prior work,^56^ whereas Δ^9^-THC (3 mg/kg s.c.) alone did not. Coadministering nonpreference-producing doses of Δ^9^-THC (3 mg/kg) and oxycodone (1 mg/kg) produced CPP in both sexes. This parallels reports that Δ^9^-THC reduces oxycodone self-administration in rats^24^^,^^57^^,^^58^—interpreted as Δ^9^-THC increasing the rewarding efficacy of a unit dose^57^^,^^58^—and that smoked cannabis increases oxycodone drug-liking in humans.^17^^,^^26^ These data raise the possibility that Δ^9^-THC coadministration could heighten reward associations with oxycodone, although Δ^9^-THC could also aid dose tapering in oxycodone dependence or with methadone^4^; either way, these reward interactions warrant consideration in any therapeutic use.

Prior reports indicate that CB1 receptors are coexpressed with *μ*-opioid receptors throughout the mesolimbic reward circuitry, and that CB1 activation can modulate dopaminergic activity in the ventral tegmental area and NAc.^21^^,^^55^ Δ^9^-THC enhances subsecond NAc dopamine release in rats,^59^ but its direct effects on accumbens dopamine in mice are subtle and dose-dependent.^60^ To evaluate a candidate dopaminergic mechanism underlying the behavioral CPP interaction, we recorded NAc dopamine release using the GRAB-DA fluorescent sensor^37^^,^^61^ following oxycodone, Δ^9^-THC, or their combination. Oxycodone produced dose-dependent NAc dopamine release that paralleled its dose-dependent CPP, whereas Δ^9^-THC alone did not produce a measurable change in NAc dopamine. Combining a subeffective oxycodone dose (1 mg/kg) with Δ^9^-THC (3 mg/kg) produced a NAc dopamine response statistically equivalent to a 3-fold higher dose of oxycodone alone (3 mg/kg). This dopamine potentiation, such as the behavioral CPP potentiation (Fig. 6, I and J), occurred at doses where each compound alone was insufficient to produce its respective effect. The potentiation was concentrated in the sustained midphase (∼15–30 minutes), consistent with the slower pharmacokinetics of subcutaneous Δ^9^-THC enhancing the sustained-phase dopamine response to oxycodone. These data indicate that systemic Δ^9^-THC may potentiate opioid-evoked NAc dopamine release at subeffective opioid doses, providing a candidate mechanism for the behavioral reward potentiation observed here and consistent with prior reports that smoked cannabis enhances oxycodone-induced drug-liking in humans.^17^

Locomotor sensitization following repeated opioid exposure has been proposed as a proxy for drug-seeking.^62^ CB1 agonists can induce catalepsy, which can confound locomotor-based assays^63^^,^^64^; however, Δ^9^-THC did not reduce locomotor activity at the dose tested here in either sex, allowing us to evaluate effects on oxycodone-induced locomotion. Coadministration of Δ^9^-THC with oxycodone did not alter oxycodone-induced hyperlocomotion or locomotor sensitization. This contrasts with a prior report in rats indicating that 41-day Δ^9^-THC pretreatment increased heroin-induced locomotor sensitization, and with the absence of opioid-induced locomotor sensitization in CB1 knockout mice,^65^ differences likely reflecting ligand and dosing conditions.

Several limitations apply. We did not test multiple dose combinations of oxycodone and Δ^9^-THC and therefore cannot definitively classify the antinociceptive interaction as synergistic versus additive; future studies incorporating dose-addition analysis are needed to fully characterize drug–drug interaction parameters. Nevertheless, our fiber photometry findings (Fig. 8) show that Δ^9^-THC potentiates oxycodone-evoked NAc dopamine release at subeffective oxycodone doses, providing mechanistic evidence consistent with a nonadditive interaction at the level of mesolimbic reward signaling. Mice in Figs. 1, 2, 6, and 7 were group-housed and received several treatment conditions, which can affect behavioral outcomes.^66^^,^^67^ Fiber photometry data were collected using a within-subjects crossover design; although sessions were spaced 2–3 days apart for washout, residual carryover effects cannot be fully excluded. Housing condition differed between the dose-response experiments (group-housed) and the combination experiments (individually housed, as required for circadian activity monitoring), and single housing is itself a stressor that can influence nociceptive responding^68^^,^^69^; housing was therefore held constant across all groups within each experiment, and no comparison in this study is made across housing conditions. Our circadian activity experiments dosed during the light cycle (when mice are less active) and used an oxycodone dose that induces hyperlocomotion.^70^ Finally, repeated hotplate testing may itself have contributed to the decline in antinociceptive magnitude across dosing days, as repeated exposure has been reported to reduce baseline latency independently of drug treatment.^71^ Vehicle controls were tested on the identical schedule in every experiment, so any such effect is shared with the comparator. We nonetheless cannot exclude a contribution of repeated testing to the antinociceptive tolerance reported here.

Our results indicate that coadministration of Δ^9^-THC with oxycodone produces a potentiation-like effect on antinociception and delays antinociceptive tolerance, although this delay was restricted to a single dose pair and the 30 minutes timepoint. The combination did not augment *μ*-opioid receptor dependence and conferred no benefit for oxycodone-induced circadian disruption. However, it produced CPP at doses that were not preference-producing alone and potentiated NAc dopamine release at an otherwise subeffective oxycodone dose. Δ^9^-THC coadministration may recruit the rewarding properties of oxycodone at otherwise subthreshold doses, so any therapeutic implementation of cannabinoid–opioid combinations must weigh a potentiation-like antinociceptive benefit against a concurrent increase in abuse liability. Future studies in the context of ongoing pain will determine whether this tradeoff is favorable.

## Conflict of interest

The authors declare no conflicts of interest.

## References

[1] Dowell D., Ragan K.R., Jones C.M., Baldwin G.T., Chou R. (2022). CDC clinical practice guideline for prescribing opioids for pain – United States, 2022. MMWR Recomm Rep.

[2] Beardsley P.M., Aceto M.D., Cook C.D., Bowman E.R., Newman J.L., Harris L.S. (2004). Discriminative stimulus, reinforcing, physical dependence, and antinociceptive effects of oxycodone in mice, rats, and rhesus monkeys. Exp Clin Psychopharmacol.

[3] Olkkola K.T., Kontinen V.K., Saari T.I., Kalso E.A. (2013). Does the pharmacology of oxycodone justify its increasing use as an analgesic?. Trends Pharmacol Sci.

[4] Strang J., Volkow N.D., Degenhardt L. (2020). Opioid use disorder. Nat Rev Dis Primers.

[5] Haroutounian S., Arendt-Nielsen L., Belton J. (2021). International association for the study of pain presidential task force on cannabis and cannabinoid analgesia: research agenda on the use of cannabinoids, cannabis, and cannabis-based medicines for pain management. Pain.

[6] Bicket M.C., Stone E.M., McGinty E.E. (2023). Use of cannabis and other pain treatments among adults with chronic pain in US states with medical cannabis programs. JAMA Netw Open.

[7] Soliman N., Haroutounian S., Hohmann A.G. (2021). Systematic review and meta-analysis of cannabinoids, cannabis-based medicines, and endocannabinoid system modulators tested for antinociceptive effects in animal models of injury-related or pathological persistent pain. Pain.

[8] Woodhams S.G., Chapman V., Finn D.P., Hohmann A.G., Neugebauer V. (2017). The cannabinoid system and pain. Neuropharmacology.

[9] Wilson-Poe A.R., Wiese B., Kibaly C. (2021). Effects of inflammatory pain on CB1 receptor in the midbrain periaqueductal gray. Pain Rep.

[10] Wilson-Poe A.R., Morgan M.M., Aicher S.A., Hegarty D.M. (2012). Distribution of CB1 cannabinoid receptors and their relationship with mu-opioid receptors in the rat periaqueductal gray. Neuroscience.

[11] Welch S.P., Stevens D.L. (1992). Antinociceptive activity of intrathecally administered cannabinoids alone, and in combination with morphine, in mice. J Pharmacol Exp Ther.

[12] Cichewicz D.L., Martin Z.L., Smith F.L., Welch S.P. (1999). Enhancement mu opioid antinociception by oral delta9-tetrahydrocannabinol: dose-response analysis and receptor identification. J Pharmacol Exp Ther.

[13] Maguire D.R., France C.P. (2018). Antinociceptive effects of mixtures of mu opioid receptor agonists and cannabinoid receptor agonists in rats: impact of drug and fixed-dose ratio. Eur J Pharmacol.

[14] Slivicki R.A., Iyer V., Mali S.S. (2020). Positive allosteric modulation of CB(1) cannabinoid receptor signaling enhances morphine antinociception and attenuates morphine tolerance without enhancing morphine- induced dependence or reward. Front Mol Neurosci.

[15] Nielsen S., Picco L., Murnion B. (2022). Opioid-sparing effect of cannabinoids for analgesia: an updated systematic review and meta-analysis of preclinical and clinical studies. Neuropsychopharmacology.

[16] Gerak L.R., Weed P.F., Maguire D.R., France C.P. (2019). Effects of the synthetic cannabinoid receptor agonist JWH-018 on abuse-related effects of opioids in rhesus monkeys. Drug Alcohol Depend.

[17] Cooper Z.D., Bedi G., Ramesh D., Balter R., Comer S.D., Haney M. (2018). Impact of co-administration of oxycodone and smoked cannabis on analgesia and abuse liability. Neuropsychopharmacology.

[18] Babalonis S., Walsh S.L. (2020). Therapeutic potential of opioid/cannabinoid combinations in humans: review of the evidence. Eur Neuropsychopharmacol.

[19] Marinello S., Powell L.M. (2023). The impact of recreational cannabis markets on motor vehicle accident, suicide, and opioid overdose fatalities. Soc Sci Med.

[20] Rhew I.C., Le V.T., Ramirez J.J. (2023). The association between cannabis use and risk of non-medical pain reliever misuse onset among young adults in a legal cannabis context. Addict Behav.

[21] Wenzel J.M., Cheer J.F. (2018). Endocannabinoid regulation of reward and reinforcement through interaction with dopamine and endogenous opioid signaling. Neuropsychopharmacology.

[22] Solinas M., Panlilio L.V., Goldberg S.R. (2004). Exposure to delta-9-tetrahydrocannabinol (THC) increases subsequent heroin taking but not heroin’s reinforcing efficacy: a self-administration study in rats. Neuropsychopharmacology.

[23] Gutierrez A., Nguyen J.D., Creehan K.M., Javadi-Paydar M., Grant Y., Taffe M.A. (2022). Effects of combined THC and heroin vapor inhalation in rats. Psychopharmacology (Berl).

[24] Nguyen J.D., Grant Y., Creehan K.M. (2019). Delta(9)-tetrahydrocannabinol attenuates oxycodone self-administration under extended access conditions. Neuropharmacology.

[25] Carey L.M., Maguire D.R., France C.P. (2023). Effects of delta9-tetrahydrocannabinol (THC), cannabidiol (CBD), and THC/CBD mixtures on fentanyl versus food choice in rhesus monkeys. Drug Alcohol Depend.

[26] Lofwall M.R., Babalonis S., Nuzzo P.A., Elayi S.C., Walsh S.L. (2016). Opioid withdrawal suppression efficacy of oral dronabinol in opioid dependent humans. Drug Alcohol Depend.

[27] Scavone J.L., Sterling R.C., Van Bockstaele E.J. (2013). Cannabinoid and opioid interactions: implications for opiate dependence and withdrawal. Neuroscience.

[28] Schlosburg J.E., Carlson B.L.A., Ramesh D. (2009). Inhibitors of endocannabinoid-metabolizing enzymes reduce precipitated withdrawal responses in THC-dependent mice. AAPS J.

[29] Bisaga A., Sullivan M.A., Glass A. (2015). The effects of dronabinol during detoxification and the initiation of treatment with extended release naltrexone. Drug Alcohol Depend.

[30] Zhang Y., Windisch K., Altschuler J., Rahm S., Butelman E.R., Kreek M.J. (2016). Adolescent oxycodone self administration alters subsequent oxycodone-induced conditioned place preference and anti-nociceptive effect in C57BL/6J mice in adulthood. Neuropharmacology.

[31] Mathis A., Mamidanna P., Cury K.M. (2018). DeepLabCut: markerless pose estimation of user-defined body parts with deep learning. Nat Neurosci.

[32] Nath T., Mathis A., Chen A.C., Patel A., Bethge M., Mathis M.W. (2019). Using DeepLabCut for 3D markerless pose estimation across species and behaviors. Nat Protoc.

[33] Slivicki R.A., Earnest T., Chang Y.H. (2023). Oral oxycodone self-administration leads to features of opioid misuse in male and female mice. Addict Biol.

[34] Goodwin N.L., Choong J.J., Hwang S. (2024). Simple Behavioral Analysis (SimBA) as a platform for explainable machine learning in behavioral neuroscience. Nat Neurosci.

[35] Land B.B., Bruchas M.R., Lemos J.C., Xu M., Melief E.J., Chavkin C. (2008). The dysphoric component of stress is encoded by activation of the dynorphin kappa-opioid system. J Neurosci.

[36] Lopes G., Bonacchi N., Frazao J. (2015). Bonsai: an event-based framework for processing and controlling data streams. Front Neuroinform.

[37] Zhuo Y., Luo B., Yi X. (2024). Improved green and red GRAB sensors for monitoring dopaminergic activity in vivo. Nat Methods.

[38] Martianova E., Aronson S., Proulx C.D. (2019). Multi-fiber photometry to record neural activity in freely-moving animals. J Vis Exp.

[39] Tallarida R.J. (2011). Quantitative methods for assessing drug synergism. Genes Cancer.

[40] Severino A.L., Mittal N., Hakimian J.K. (2020). μ-opioid receptors on distinct neuronal populations mediate different aspects of opioid reward-related behaviors. eNeuro.

[41] Wakley A.A., Wiley J.L., Craft R.M. (2014). Sex differences in antinociceptive tolerance to delta-9-tetrahydrocannabinol in the rat. Drug Alcohol Depend.

[42] Henderson-Redmond A.N., Sepulveda D.E., Ferguson E.L., Kline A.M., Piscura M.K., Morgan D.J. (2022). Sex-specific mechanisms of tolerance for the cannabinoid agonists CP55,940 and delta-9-tetrahydrocannabinol (Delta(9)-THC). Psychopharmacology (Berl).

[43] Cooper Z.D., Craft R.M. (2018). Sex-dependent effects of cannabis and cannabinoids: a translational perspective. Neuropsychopharmacology.

[44] Collins D., Reed B., Zhang Y., Kreek M.J. (2016). Sex differences in responsiveness to the prescription opioid oxycodone in mice. Pharmacol Biochem Behav.

[45] Tagne A.M., Fotio Y., Gupta K., Piomelli D. (2024). Δ^9^-Tetrahydrocannabinol alleviates hyperalgesia in a humanized mouse model of sickle cell disease. J Pharmacol Exp Ther.

[46] Williams I.J., Edwards S., Rubo A., Haller V.L., Stevens D.L., Welch S.P. (2006). Time course of the enhancement and restoration of the analgesic efficacy of codeine and morphine by delta9-tetrahydrocannabinol. Eur J Pharmacol.

[47] Gerak L.R., France C.P. (2016). Combined treatment with morphine and delta9-tetrahydrocannabinol in rhesus monkeys: antinociceptive tolerance and withdrawal. J Pharmacol Exp Ther.

[48] van Dam C.J., van Velzen M., Kramers C. (2023). Cannabis-opioid interaction in the treatment of fibromyalgia pain: an open-label, proof of concept study with randomization between treatment groups: cannabis, oxycodone or cannabis/oxycodone combination-the SPIRAL study. Trials.

[49] Enga R.M., Jackson A., Damaj M.I., Beardsley P.M. (2016). Oxycodone physical dependence and its oral self-administration in C57BL/6J mice. Eur J Pharmacol.

[50] Carper M., Contreras K.M., Walentiny D.M., Beardsley P.M., Damaj M.I. (2021). Validation and characterization of oxycodone physical dependence in C57BL/6J mice. Eur J Pharmacol.

[51] Gamble M.C., Chuan B., Gallego-Martin T. (2022). A role for the circadian transcription factor NPAS2 in the progressive loss of non-rapid eye movement sleep and increased arousal during fentanyl withdrawal in male mice. Psychopharmacology (Berl).

[52] Marchant E.G., Mistlberger R.E. (1995). Morphine phase-shifts circadian rhythms in mice: role of behavioural activation. Neuroreport.

[53] Kesner A.J., Mateo Y., Abrahao K.P. (2022). Changes in striatal dopamine release, sleep, and behavior during spontaneous Delta-9-tetrahydrocannabinol abstinence in male and female mice. Neuropsychopharmacology.

[54] Iyer V., Rangel-Barajas C., Woodward T.J. (2022). Negative allosteric modulation of CB(1) cannabinoid receptor signaling suppresses opioid-mediated reward. Pharmacol Res.

[55] Mohammadkhani A., Borgland S.L. (2022). Cellular and behavioral basis of cannabinioid and opioid interactions: implications for opioid dependence and withdrawal. J Neurosci Res.

[56] Niikura K., Ho A., Kreek M.J., Zhang Y. (2013). Oxycodone-induced conditioned place preference and sensitization of locomotor activity in adolescent and adult mice. Pharmacol Biochem Behav.

[57] Nguyen J.D., Grant Y., Taffe M.A. (2021). Paradoxical changes in brain reward status during oxycodone self-administration in a novel test of the negative reinforcement hypothesis. Br J Pharmacol.

[58] Nguyen J.D., Grant Y., Yang C., Gutierrez A., Taffe M.A. (Posted online October 31, 2023). Oxycodone self-administration in female rats is enhanced by Δ^9^-tetrahydrocannabinol, but not by cannabidiol, in a progressive ratio procedure. bioRxiv.

[59] Cheer J.F., Wassum K.M., Heien M.L.A.V., Phillips P.E.M., Wightman R.M. (2004). Cannabinoids enhance subsecond dopamine release in the nucleus accumbens of awake rats. J Neurosci.

[60] Bloomfield M.A.P., Ashok A.H., Volkow N.D., Howes O.D. (2016). The effects of Δ^9^-tetrahydrocannabinol on the dopamine system. Nature.

[61] Sun F., Zhou J., Dai B. (2020). Next-generation GRAB sensors for monitoring dopaminergic activity in vivo. Nat Methods.

[62] Robinson T.E., Berridge K.C. (1993). The neural basis of drug craving: an incentive-sensitization theory of addiction. Brain Res Brain Res Rev.

[63] Metna-Laurent M., Mondesir M., Grel A., Vallee M., Piazza P.-V. (2017). Cannabinoid-induced tetrad in mice. Curr Protoc Neurosci.

[64] Slivicki R.A., Yi J., Brings V.E., Huynh P.N., Gereau R.W. (2022). The cannabinoid agonist CB-13 produces peripherally mediated analgesia in mice but elicits tolerance and signs of central nervous system activity with repeated dosing. Pain.

[65] Martin M., Ledent C., Parmentier M., Maldonado R., Valverde O. (2000). Cocaine, but not morphine, induces conditioned place preference and sensitization to locomotor responses in CB1 knockout mice. Eur J Neurosci.

[66] Bates M.L.S., Emery M.A., Wellman P.J., Eitan S. (2016). Social environment alters opioid-induced hyperalgesia and antinociceptive tolerance in adolescent mice. Eur J Pain.

[67] de Oliveira P.E.C., Carmona I.M., Casarotto M., Silveira L.M., Oliveira A.C.B., Canto-de-Souza A. (2022). Mice cohabiting with familiar conspecific in chronic stress condition exhibit methamphetamine-induced locomotor sensitization and augmented consolation behavior. Front Behav Neurosci.

[68] Pitzer C., Kuner R., Tappe-Theodor A. (2016). Voluntary and evoked behavioral correlates in inflammatory pain conditions under different social housing conditions. Pain Rep.

[69] Würbel H. (2001). Ideal homes? Housing effects on rodent brain and behaviour. Trends Neurosci.

[70] Brings V.E., Payne M.A., Gereau R.W. (2022). Opioids alter paw placement during walking, confounding assessment of analgesic efficacy in a postsurgical pain model in mice. Pain Rep.

[71] Gunn A., Bobeck E.N., Weber C., Morgan M.M. (2011). The influence of non-nociceptive factors on hot-plate latency in rats. J Pain.

