# Supplementary material for: Impact of Δ9-tetrahydrocannabinol and oxycodone coadministration on measures of antinociception, dependence, circadian activity, and reward in mice
Source: J Pharmacol Exp Ther. 2026 Aug 14;393(9):105008. doi: 10.1016/j.jpet.2026.105008 (PMC13688984; doi:10.1016/j.jpet.2026.105008)
Supplement: Supplementary file 1 [file mmc1.pdf]

Impact of Δ^9-tetrahydrocannabinol and oxycodone co-administration on measures of antinociception, dependence, circadian activity, and reward in mice

Richard A. Slivicki[1], Justin G. Wang[1,2], Kayla V. Kucz[1], Jakayla Folarin Hines[1], Vy Trinh Tran Nhat[1], Alexxai V. Kravitz[1,3,4], Meaghan C. Creed[1,4] and Robert W. Gereau IV[1,4,5]

[1]Washington University Pain Center and Department of Anesthesiology, [2]Neuroscience Graduate Program, Division of Biology & Biomedical Sciences, [3]Department of Psychiatry, [4]Department of Neuroscience, [5]Department of Biomedical Engineering, Washington University, St. Louis, MO

Journal of Pharmacology and Experimental Therapeutics

Supplemental Material

Contents

Supplemental Figures 1–3
Supplemental Tables 1–8

Supplemental Figure 1

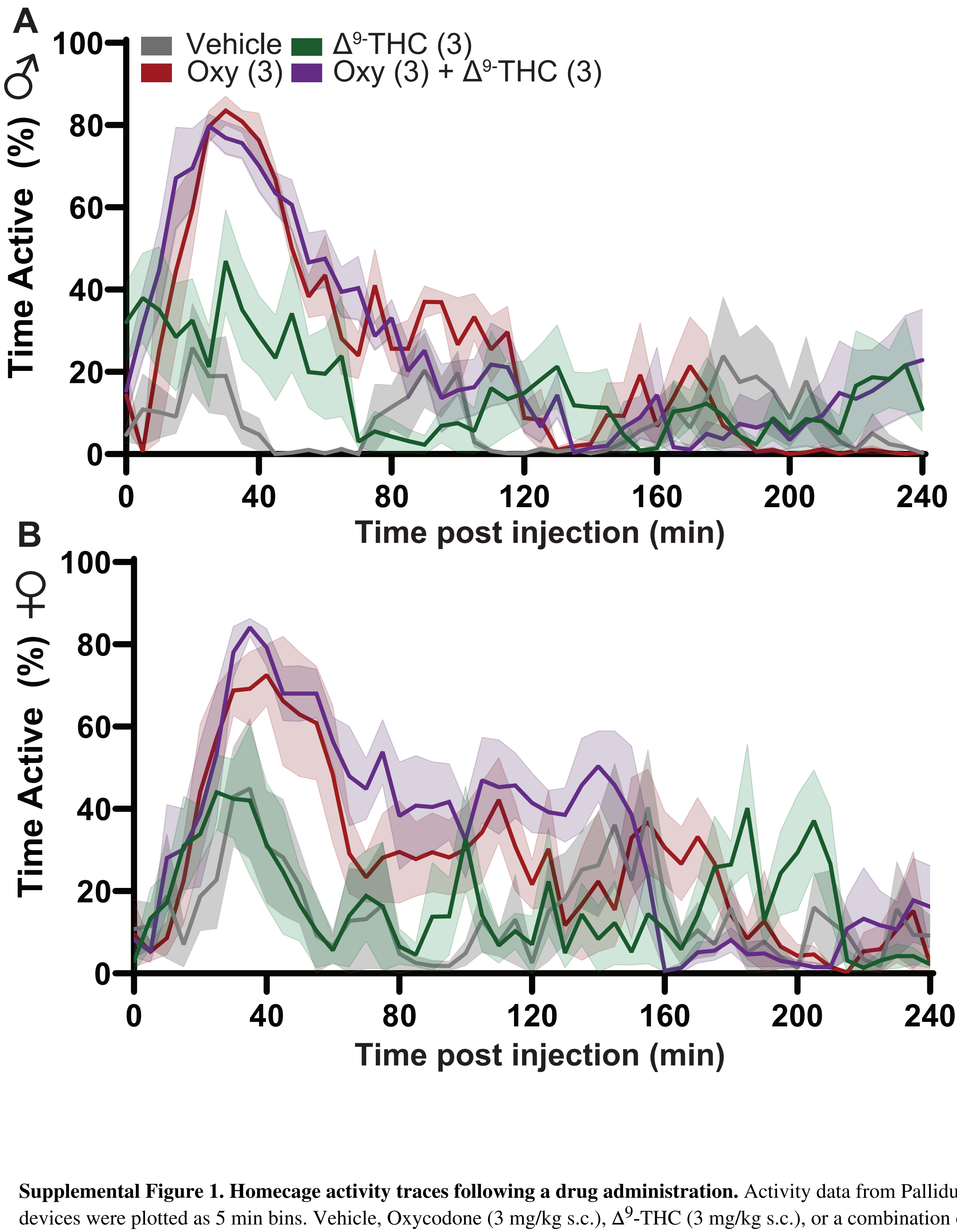

Supplemental Figure 1. Homecage activity traces following a drug administration. Activity data from Pallidus devices were plotted as 5 min bins. Vehicle, Oxycodone (3 mg/kg s.c.), Δ^9-THC (3 mg/kg s.c.), or a combination of oxycodone and Δ^9-THC was administered at the 0 min mark in both males (A) and females (B). Oxycodone and combination treatment of oxycodone and Δ^9-THC produced transient increases in activity in both males and females. Data are represented as mean ± SEM. N = 5-6 per group.

Supplemental Figure 2

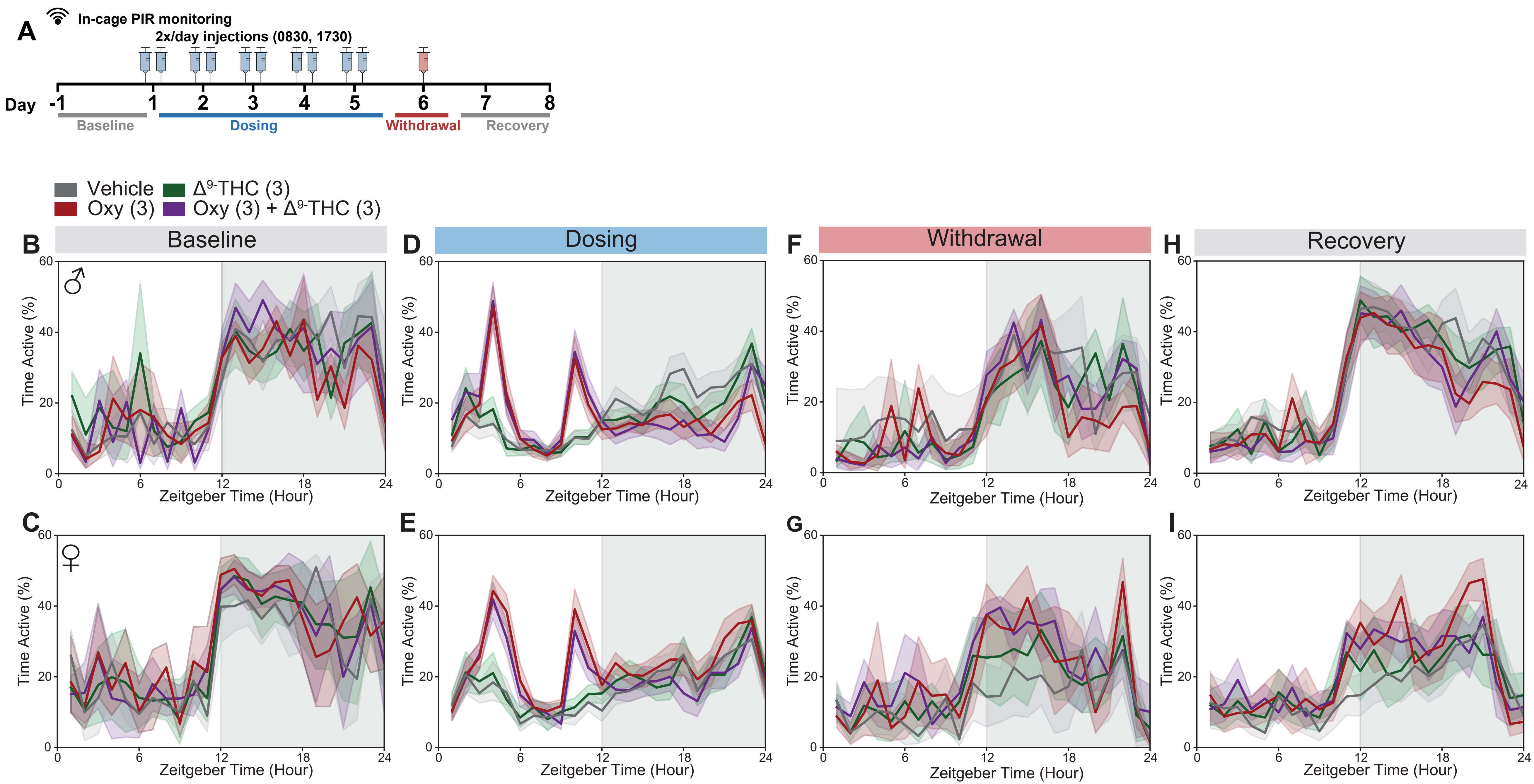

Supplemental Figure 2. Oxycodone and combination of Δ^9-THC with oxycodone produce alterations in circadian activity during the dosing phase. Schematic of experimental timecourse (A). Activity data from Pallidus devices were plotted as 1 hr bins and averaged for each phase of the experiment. Activity was similar across all groups during the baseline phase of the experiment in both males (B) and females (C). During the dosing phase, treatment with oxycodone and a combination of oxycodone and Δ^9-THC produced a spike in activity during the light phase in both males (D) and females (E) that corresponded to the time when the treatment was administered. There were slight differences between groups during the withdrawal phase (F,G) and groups appeared mostly similar during the recovery phase (H,I). Data are represented as mean ± SEM. N = 5 per group.

Supplemental Figure 3

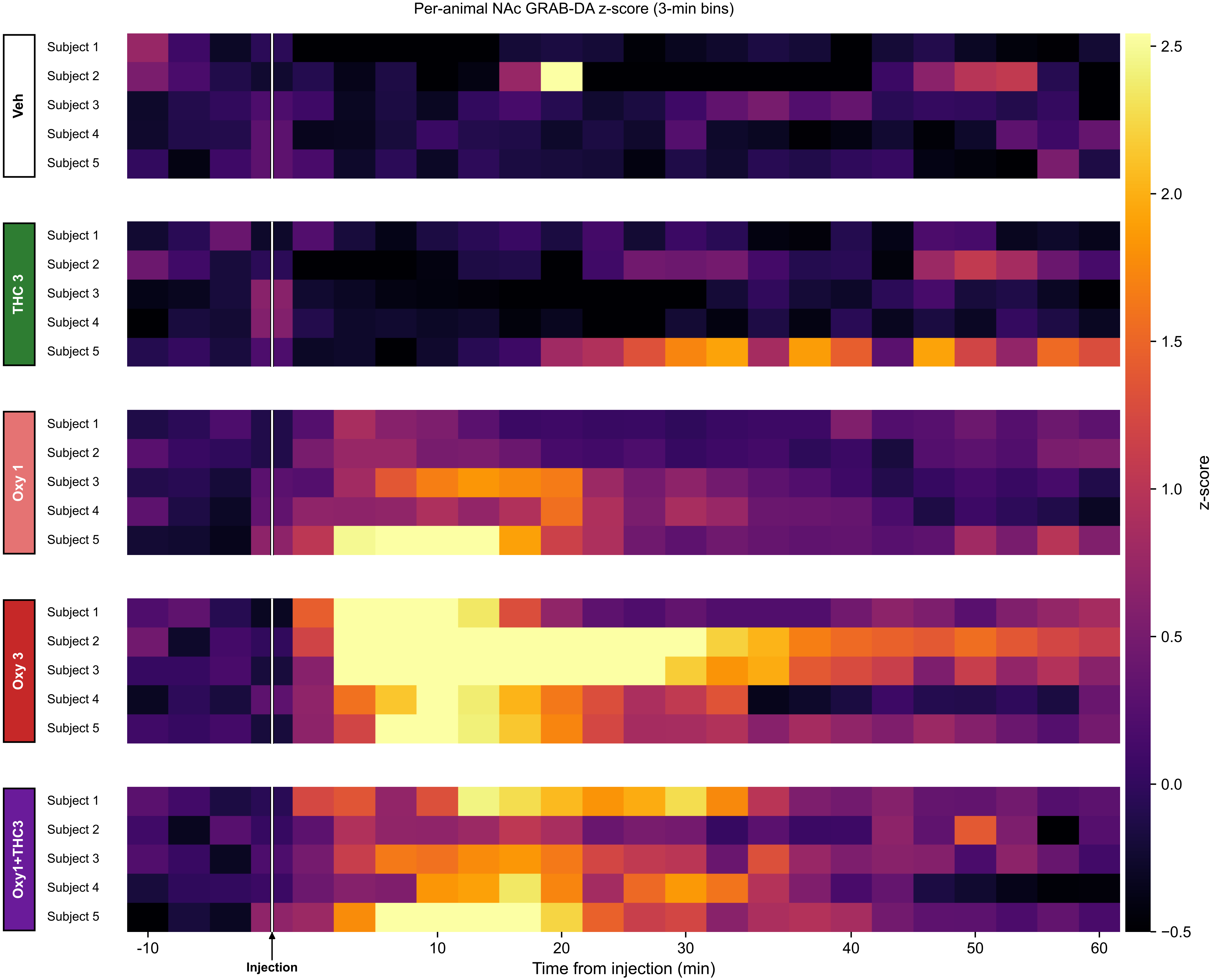

Supplemental Figure 3. Per-animal NAc GRAB-DA z-score time courses, sorted by AUC within condition. Heatmap of per-animal z-score time courses (rows = individual animals; columns = 3-min bins from –10 to +60 min relative to injection; color = z-score against the pre-injection baseline). Animals are grouped by treatment condition (color labels at right) and sorted within condition by descending post-injection AUC. The dashed black line marks injection time (t = 0). Vehicle and Δ^9-THC traces are uniformly near baseline; Oxycodone 1 mg/kg shows a modest 0–15 minute response in cohort 1 high responders; Oxycodone 3 mg/kg shows robust early elevation in most animals; the oxycodone (1 mg/kg) + Δ^9-THC (3 mg/kg) condition shows a sustained 5–30 minute elevation in all animals. N = 5 per group.

Supplemental Statistical Tables

Slivicki et al., Impact of Δ⁹-tetrahydrocannabinol and oxycodone co-administration on measures of antinociception, dependence, circadian activity, and reward in mice

Supplemental Table 1. Statistics from Figure 1

Figure 1D – Male Day 1

Type of test	Comparison	P-value summary	F, P-value
Two-way ANOVA	Time	****	F (3.548, 88.70) = 56.29, P<0.0001
	Treatment	****	F (4, 25) = 15.83, P<0.0001
	Interaction	****	F (20, 125) = 10.41, P<0.0001
Tukey's post-hoc			
30	Vehicle vs. 1 mg/kg	ns	0.9732
	Vehicle vs. 3 mg/kg	*	0.0332
	Vehicle vs. 10 mg/kg	***	0.001
	Vehicle vs. 30 mg/kg	****	<0.0001
	1 mg/kg vs. 3 mg/kg	ns	0.105
	1 mg/kg vs. 10 mg/kg	**	0.0049
	1 mg/kg vs. 30 mg/kg	**	0.0013
	3 mg/kg vs. 10 mg/kg	ns	0.5593
	3 mg/kg vs. 30 mg/kg	*	0.0415
	10 mg/kg vs. 30 mg/kg	ns	0.1816
60	Vehicle vs. 1 mg/kg	ns	0.8172
	Vehicle vs. 3 mg/kg	ns	0.3981
	Vehicle vs. 10 mg/kg	**	0.0013
	Vehicle vs. 30 mg/kg	****	<0.0001
	1 mg/kg vs. 3 mg/kg	ns	>0.9999
	1 mg/kg vs. 10 mg/kg	*	0.0245
	1 mg/kg vs. 30 mg/kg	**	0.0036
	3 mg/kg vs. 10 mg/kg	**	0.005
	3 mg/kg vs. 30 mg/kg	****	<0.0001
	10 mg/kg vs. 30 mg/kg	ns	0.3323
90	Vehicle vs. 1 mg/kg	ns	0.7073
	Vehicle vs. 3 mg/kg	ns	0.8368
	Vehicle vs. 10 mg/kg	ns	0.3137
	Vehicle vs. 30 mg/kg	*	0.0131
	1 mg/kg vs. 3 mg/kg	ns	>0.9999
	1 mg/kg vs. 10 mg/kg	ns	0.7778
	1 mg/kg vs. 30 mg/kg	*	0.0238
	3 mg/kg vs. 10 mg/kg	ns	0.8368
	3 mg/kg vs. 30 mg/kg	*	0.022
	10 mg/kg vs. 30 mg/kg	ns	0.0676

Figure 1E – Female Day 1

Type of test	Comparison	P-value summary	F, P-value
Two-way ANOVA	Time	****	F (3.075, 61.51) = 31.72, P<0.0001
	Treatment	****	F (4, 20) = 13.42, P<0.0001

Type of test	Comparison	P-value summary	F, P-value
	Interaction	****	F (20, 100) = 10.18, P<0.0001
Tukey's post-hoc			
30	Vehicle vs. 1 mg/kg	ns	0.9807
	Vehicle vs. 3 mg/kg	*	0.0179
	Vehicle vs. 10 mg/kg	**	0.0063
	Vehicle vs. 30 mg/kg	****	<0.0001
	1 mg/kg vs. 3 mg/kg	ns	0.1141
	1 mg/kg vs. 10 mg/kg	**	0.0078
	1 mg/kg vs. 30 mg/kg	***	0.0001
	3 mg/kg vs. 10 mg/kg	ns	0.0652
	3 mg/kg vs. 30 mg/kg	***	0.0005
	10 mg/kg vs. 30 mg/kg	ns	0.6525
60	Vehicle vs. 1 mg/kg	ns	0.9657
	Vehicle vs. 3 mg/kg	ns	0.8371
	Vehicle vs. 10 mg/kg	ns	0.165
	Vehicle vs. 30 mg/kg	***	0.0009
	1 mg/kg vs. 3 mg/kg	ns	0.9412
	1 mg/kg vs. 10 mg/kg	ns	0.1009
	1 mg/kg vs. 30 mg/kg	****	<0.0001
	3 mg/kg vs. 10 mg/kg	ns	0.3917
	3 mg/kg vs. 30 mg/kg	***	0.0003
	10 mg/kg vs. 30 mg/kg	**	0.002

Figure 1F – Male Day 3

Type of test	Comparison	P-value summary	F, P-value
Two-way ANOVA	Time	****	F (3.057, 76.44) = 10.15, P<0.0001
	Treatment	ns	F (4, 25) = 1.724, P=0.1763
	Interaction	***	F (20, 125) = 2.689, P=0.0005
Tukey's post-hoc			
30	Vehicle vs. 1 mg/kg	ns	0.0702
	Vehicle vs. 3 mg/kg	ns	0.593
	Vehicle vs. 10 mg/kg	ns	0.1449
	Vehicle vs. 30 mg/kg	***	0.0009
	1 mg/kg vs. 3 mg/kg	ns	>0.9999
	1 mg/kg vs. 10 mg/kg	ns	0.9477
	1 mg/kg vs. 30 mg/kg	*	0.0177
	3 mg/kg vs. 10 mg/kg	ns	0.9898
	3 mg/kg vs. 30 mg/kg	ns	0.0772
	10 mg/kg vs. 30 mg/kg	ns	0.0504

Figure 1G – Female Day 3

Type of test	Comparison	P-value summary	F, P-value
Two-way ANOVA	Time	ns	F (3.563, 71.26) = 2.259, P=0.0784
	Treatment	ns	F (4, 20) = 1.248, P=0.3228
	Interaction	*	F (20, 100) = 1.974, P=0.0148
Tukey's post-hoc			
	No post-hoc comparison reached significance at any timepoint.		

Figure 1H – Male Day 5

Type of test	Comparison	P-value summary	F, P-value
Two-way ANOVA	Time	*	F (4.136, 103.4) = 2.826, P=0.0271
	Treatment	ns	F (4, 25) = 0.3391, P=0.8489
	Interaction	***	F (20, 125) = 2.756, P=0.0003
Tukey's post-hoc			
	No post-hoc comparison reached significance at any timepoint.		

Figure 1I – Female Day 5

Type of test	Comparison	P-value summary	F, P-value
Two-way ANOVA	Time	**	F (3.477, 69.54) = 5.127, P=0.0018
	Treatment	ns	F (4, 20) = 1.873, P=0.1547
	Interaction	*	F (20, 100) = 1.690, P=0.0475
Tukey's post-hoc			
	No post-hoc comparison reached significance at any timepoint.		

Supplemental Table 2. Statistics from Figure 2

Figure 2D – Male Day 1

Type of test	Comparison	P-value summary	F, P-value
Two-way ANOVA	Time	****	F (3.282, 82.04) = 14.18, P<0.0001
	Treatment	****	F (4, 25) = 13.06, P<0.0001
	Interaction	****	F (20, 125) = 3.948, P<0.0001
Tukey's post-hoc			
30	1 mg/kg vs. 3 mg/kg	ns	0.2789
	1 mg/kg vs. 10 mg/kg	*	0.0448
	1 mg/kg vs. 30 mg/kg	**	0.001
	Vehicle vs. 1 mg/kg	ns	0.9015
	3 mg/kg vs. 10 mg/kg	ns	0.4916
	3 mg/kg vs. 30 mg/kg	*	0.0217
	Vehicle vs. 3 mg/kg	ns	0.0899
	10 mg/kg vs. 30 mg/kg	ns	0.5304
	Vehicle vs. 10 mg/kg	*	0.0181
	Vehicle vs. 30 mg/kg	***	0.0004
60	1 mg/kg vs. 3 mg/kg	ns	0.1214
	1 mg/kg vs. 10 mg/kg	ns	0.1076
	1 mg/kg vs. 30 mg/kg	**	0.0021
	Vehicle vs. 1 mg/kg	ns	0.9369
	3 mg/kg vs. 10 mg/kg	ns	0.7871
	3 mg/kg vs. 30 mg/kg	*	0.0286
	Vehicle vs. 3 mg/kg	ns	0.0732
	10 mg/kg vs. 30 mg/kg	ns	0.3627
	Vehicle vs. 10 mg/kg	ns	0.1706
	Vehicle vs. 30 mg/kg	**	0.0027
90	1 mg/kg vs. 3 mg/kg	ns	0.2139
	1 mg/kg vs. 10 mg/kg	ns	0.1909
	1 mg/kg vs. 30 mg/kg	*	0.0452

Type of test	Comparison	P-value summary	F, P-value
	Vehicle vs. 1 mg/kg	ns	0.9979
	3 mg/kg vs. 10 mg/kg	ns	>0.9999
	3 mg/kg vs. 30 mg/kg	ns	0.6894
	Vehicle vs. 3 mg/kg	ns	0.2292
	10 mg/kg vs. 30 mg/kg	ns	0.7628
	Vehicle vs. 10 mg/kg	ns	0.2059
	Vehicle vs. 30 mg/kg	ns	0.0516
180	1 mg/kg vs. 3 mg/kg	ns	0.9922
	1 mg/kg vs. 10 mg/kg	ns	0.9296
	1 mg/kg vs. 30 mg/kg	ns	0.0834
	Vehicle vs. 1 mg/kg	ns	0.9504
	3 mg/kg vs. 10 mg/kg	ns	0.8474
	3 mg/kg vs. 30 mg/kg	ns	0.0618
	Vehicle vs. 3 mg/kg	ns	0.9989
	10 mg/kg vs. 30 mg/kg	ns	0.443
	Vehicle vs. 10 mg/kg	ns	0.7701
	Vehicle vs. 30 mg/kg	*	0.0494

Figure 2E – Female Day 1

Type of test	Comparison	P-value summary	F, P-value
Two-way ANOVA	Time	****	F (3.084, 61.67) = 12.24, P<0.0001
	Treatment	***	F (4, 20) = 7.246, P=0.0009
	Interaction	****	F (20, 100) = 3.574, P<0.0001
Tukey's post-hoc			
30	Vehicle vs. 1 mg/kg	ns	0.9158
	Vehicle vs. 3 mg/kg	**	0.0065
	Vehicle vs. 10 mg/kg	*	0.0206
	Vehicle vs. 30 mg/kg	*	0.0465
	1 mg/kg vs. 3 mg/kg	ns	0.1389
	1 mg/kg vs. 10 mg/kg	ns	0.1345
	1 mg/kg vs. 30 mg/kg	ns	0.0749
	3 mg/kg vs. 10 mg/kg	ns	0.9949
	3 mg/kg vs. 30 mg/kg	ns	0.4126
	10 mg/kg vs. 30 mg/kg	ns	0.5386

Figure 2F – Male Day 3

Type of test	Comparison	P-value summary	F, P-value
Two-way ANOVA	Time	**	F (3.466, 86.64) = 4.806, P=0.0025
	Treatment	ns	F (4, 25) = 2.419, P=0.0751
	Interaction	ns	F (20, 125) = 0.9019, P=0.5853
Tukey's post-hoc			
30	Vehicle vs. 1 mg/kg	ns	0.9355
	Vehicle vs. 3 mg/kg	ns	0.7759
	Vehicle vs. 10 mg/kg	*	0.0197
	Vehicle vs. 30 mg/kg	ns	0.1948
	1 mg/kg vs. 3 mg/kg	ns	0.9446
	1 mg/kg vs. 10 mg/kg	*	0.0281
	1 mg/kg vs. 30 mg/kg	ns	0.3097
	3 mg/kg vs. 10 mg/kg	ns	0.6231

Type of test	Comparison	P-value summary	F, P-value
	3 mg/kg vs. 30 mg/kg	ns	0.7753
	10 mg/kg vs. 30 mg/kg	ns	>0.9999

Figure 2G – Female Day 3

Type of test	Comparison	P-value summary	F, P-value
Two-way ANOVA	Time	*	F (3.362, 63.87) = 3.100, P=0.0280
	Treatment	ns	F (4, 19) = 0.9639, P=0.4499
	Interaction	ns	F (20, 95) = 1.652, P=0.0563
Tukey's post-hoc			
60	Vehicle vs. 1 mg/kg	ns	0.9693
	Vehicle vs. 3 mg/kg	*	0.0179
	Vehicle vs. 10 mg/kg	ns	0.692
	Vehicle vs. 30 mg/kg	ns	0.1883
	1 mg/kg vs. 3 mg/kg	ns	0.8628
	1 mg/kg vs. 10 mg/kg	ns	0.9993
	1 mg/kg vs. 30 mg/kg	ns	0.9871
	3 mg/kg vs. 10 mg/kg	ns	0.778
	3 mg/kg vs. 30 mg/kg	ns	0.7483
	10 mg/kg vs. 30 mg/kg	ns	0.9961

Figure 2H – Male Day 5

Type of test	Comparison	P-value summary	F, P-value
Two-way ANOVA	Time	ns	F (4.051, 101.3) = 0.5761, P=0.6828
	Treatment	ns	F (4, 25) = 1.967, P=0.1305
	Interaction	ns	F (16.21, 101.3) = 0.7048, P=0.7850
Tukey's post-hoc			
	No post-hoc comparison reached significance at any timepoint.		

Figure 2I – Female Day 5

Type of test	Comparison	P-value summary	F, P-value
Two-way ANOVA	Time	ns	F (3.829, 72.75) = 1.438, P=0.2318
	Treatment	ns	F (4, 19) = 2.776, P=0.0569
	Interaction	ns	F (20, 95) = 0.8604, P=0.6356
Tukey's post-hoc			
30	Vehicle vs. 1 mg/kg	ns	0.9877
	Vehicle vs. 3 mg/kg	ns	>0.9999
	Vehicle vs. 10 mg/kg	ns	0.8824
	Vehicle vs. 30 mg/kg	*	0.04
	1 mg/kg vs. 3 mg/kg	ns	0.9956
	1 mg/kg vs. 10 mg/kg	ns	0.7148
	1 mg/kg vs. 30 mg/kg	*	0.0167
	3 mg/kg vs. 10 mg/kg	ns	0.9388
	3 mg/kg vs. 30 mg/kg	ns	0.1567
	10 mg/kg vs. 30 mg/kg	ns	0.515
90	Vehicle vs. 1 mg/kg	ns	0.7097
	Vehicle vs. 3 mg/kg	ns	0.9563

Type of test	Comparison	P-value summary	F, P-value
	Vehicle vs. 10 mg/kg	ns	0.9829
	Vehicle vs. 30 mg/kg	ns	0.6639
	1 mg/kg vs. 3 mg/kg	ns	0.856
	1 mg/kg vs. 10 mg/kg	ns	0.813
	1 mg/kg vs. 30 mg/kg	ns	0.1676
	3 mg/kg vs. 10 mg/kg	ns	0.9995
	3 mg/kg vs. 30 mg/kg	*	0.0353
	10 mg/kg vs. 30 mg/kg	ns	0.0873

Supplemental Table 3. Statistics from Figure 3

Figure 3B – Male day 1

Type of test	Comparison	P-value summary	F, P-value
Two-way ANOVA	Time	****	F (2.902, 46.43) = 21.78, P<0.0001
	Treatment	****	F (3, 16) = 18.68, P<0.0001
	Interaction	****	F (15,80) = 6.968, P<0.0001
Tukey's post-hoc			
30	Vehicle vs. Δ^9-THC (3)	ns	0.9475
	Vehicle vs. Oxycodone (3)	**	0.0038
	Vehicle vs. Δ^9-THC (3) + Oxy (3)	***	0.0002
	Δ^9-THC (3) vs. Oxycodone (3)	**	0.0057
	Δ^9-THC (3) vs. Δ^9-THC (3) + Oxy (3)	***	0.0003
	Oxycodone (3) vs. Δ^9-THC (3) + Oxy (3)	*	0.0111
	Vehicle vs. Δ^9-THC (3)	**	0.001
60	Vehicle vs. Δ^9-THC (3)	ns	0.9523
	Vehicle vs. Oxycodone (3)	ns	0.7792
	Vehicle vs. Δ^9-THC (3) + Oxy (3)	*	0.0296
	Δ^9-THC (3) vs. Oxycodone (3)	ns	0.6429
	Δ^9-THC (3) vs. Δ^9-THC (3) + Oxy (3)	*	0.0195
	Oxycodone (3) vs. Δ^9-THC (3) + Oxy (3)	ns	0.0513

Figure 3C – Female day 1

Type of test	Comparison	P-value summary	F, P-value
Two-way ANOVA	Time	****	F (2.470, 39.51) = 18.72, P<0.0001
	Treatment	****	F (3, 16) = 5.458, P<0.0001
	Interaction	****	F (15,80) = 5.596, P<0.0001
Tukey's post-hoc			
30	Vehicle vs. Δ^9-THC (3)	ns	0.1566
	Vehicle vs. Oxycodone (3)	ns	0.0938
	Vehicle vs. Δ^9-THC (3) + Oxy (3)	**	0.0016
	Δ^9-THC (3) vs. Oxycodone (3)	ns	0.8679
	Δ^9-THC (3) vs. Δ^9-THC (3) + Oxy (3)	**	0.0061

Type of test	Comparison	P-value summary	F, P-value
	Oxycodone (3) vs. Δ^9-THC (3) + Oxy (3)	*	0.0108
	Vehicle vs. Δ^9-THC (3)	ns	0.1566

Figure 3D – Male day 3

Type of test	Comparison	P-value summary	F, P-value
Two-way ANOVA	Time	**	F (2.885, 46.16) = 18.72, P=0.0019
	Treatment	**	F (3, 16) = 5.605, P=0.0080
	Interaction	****	F (15,80) = 4.194, P<0.0001
Tukey's post-hoc			
30	Vehicle vs. Δ^9-THC (3)	ns	0.7055
	Vehicle vs. Oxycodone (3)	ns	0.4755
	Vehicle vs. Δ^9-THC (3) + Oxy (3)	**	0.0025
	Δ^9-THC (3) vs. Oxycodone (3)	ns	0.9616
	Δ^9-THC (3) vs. Δ^9-THC (3) + Oxy (3)	*	0.011
	Oxycodone (3) vs. Δ^9-THC (3) + Oxy (3)	*	0.0295
	Vehicle vs. Δ^9-THC (3)	ns	0.7055

Figure 3E – Female day 3

Type of test	Comparison	P-value summary	F, P-value
Two-way ANOVA	Time	****	F (3.393, 54.29) = 10.92, P<0.0001
	Treatment	ns	F (3, 16) = 5.605, P=0.0080
	Interaction	***	F (15,80) = 3.268, P<0.001
Tukey's post-hoc			
30	Vehicle vs. Δ^9-THC (3)	ns	0.9999
	Vehicle vs. Oxycodone (3)	ns	0.9999
	Vehicle vs. Δ^9-THC (3) + Oxy (3)	*	0.0253
	Δ^9-THC (3) vs. Oxycodone (3)	ns	>0.9999
	Δ^9-THC (3) vs. Δ^9-THC (3) + Oxy (3)	ns	0.0566
	Oxycodone (3) vs. Δ^9-THC (3) + Oxy (3)	*	0.0264
	Vehicle vs. Δ^9-THC (3)	ns	0.9999

Supplemental Table 4. Statistics from Figure 4

Figure 4B – Oxycodone Withdrawal Males

Type of test	Comparison	P-value summary	F, P-value
One-way ANOVA	All groups	****	F (4, 24) = 5.039, P=0.0043
Tukey's post-hoc	0 vs. 1	ns	0.2972
	0 vs. 3	ns	0.0577
	0 vs. 10	*	0.0389
	0 vs. 30	**	0.0024
	1 vs. 3	ns	0.9009

Type of test	Comparison	P-value summary	F, P-value
	1 vs. 10	ns	0.8225
	1 vs. 30	ns	0.1687
	3 vs. 10	ns	0.9997
	3 vs. 30	ns	0.5759
	10 vs. 30	ns	0.6821

Figure 4C – Oxycodone Withdrawal Females

Type of test	Comparison	P-value summary	F, P-value
One-way ANOVA	All groups	****	F (4, 20) = 4.692, P=0.0078
Tukey's post-hoc	0 vs. 1	ns	0.7539
	0 vs. 3	ns	0.724
	0 vs. 10	*	0.0447
	0 vs. 30	*	0.0243
	1 vs. 3	ns	>0.9999
	1 vs. 10	ns	0.312
	1 vs. 30	ns	0.1904
	3 vs. 10	ns	0.3387
	3 vs. 30	ns	0.2093
	10 vs. 30	ns	0.9978

Figure 4D – Oxy + THC Withdrawal Males

Type of test	Comparison	P-value summary	F, P-value
One-way ANOVA	All groups	**	F (3, 16) = 7.853, P=0.0019
Tukey's post-hoc	Vehicle vs. Δ^9-THC (3)	ns	>0.9999
	Vehicle vs. Oxy (3)	**	0.0034
	Vehicle vs. Oxy (3) +THC (3)	ns	0.5582
	Δ^9-THC (3) vs. Oxy (3)	**	0.0034
	Δ^9-THC (3) vs. Oxy (3) +THC (3)	ns	0.5582
	Oxy(3) vs. Oxy(3) +THC (3)	*	0.0489

Figure 4E – Oxy + THC Withdrawal Females

Type of test	Comparison	P-value summary	F, P-value
One-way ANOVA	All groups	**	F (3, 16) = 5.300, P=0.0099
Tukey's post-hoc	Vehicle vs. Δ^9-THC (3)	ns	0.999
	Vehicle vs. Oxy (3)	ns	0.0794
	Vehicle vs. Oxy (3) +THC (3)	*	0.0493
	Δ^9-THC (3) vs. Oxy (3)	ns	0.0611
	Δ^9-THC (3) vs. Oxy (3) +THC (3)	*	0.0376
	Oxy(3) vs. Oxy(3) +THC (3)	ns	0.9941

Supplemental Table 5. Statistics from Figure 5

Figure 5G – Circadian Activity Index Males

Type of test	Comparison	P-value summary	F, P-value
Two-way ANOVA	Time	****	F (2.568, 41.09) = 87.21, P<0.0001
	Treatment	****	F (3, 16) = 15.52, P<0.0001
	Interaction	****	F (9,48) = 7.326, P<0.0001
Tukey's post-hoc			
Baseline	Vehicle vs. Δ^9-THC (3)	ns	0.639
	Vehicle vs. Oxycodone (3)	ns	0.8054
	Vehicle vs. Δ^9-THC (3) + Oxy (3)	ns	0.9986
	Δ^9-THC (3) vs. Oxycodone (3)	ns	0.9975
	Δ^9-THC (3) vs. Δ^9-THC (3) + Oxy (3)	ns	0.4463
	Oxycodone (3) vs. Δ^9-THC (3) + Oxy (3)	ns	0.7425
Dosing	Vehicle vs. Δ^9-THC (3)	ns	0.0747
	Vehicle vs. Oxycodone (3)	**	0.0018
	Vehicle vs. Δ^9-THC (3) + Oxy (3)	****	<0.0001
	Δ^9-THC (3) vs. Oxycodone (3)	*	0.0127
	Δ^9-THC (3) vs. Δ^9-THC (3) + Oxy (3)	***	0.0008
	Oxycodone (3) vs. Δ^9-THC (3) + Oxy (3)	ns	0.5293
Withdrawal	Vehicle vs. Δ^9-THC (3)	ns	0.9893
	Vehicle vs. Oxycodone (3)	ns	0.0771
	Vehicle vs. Δ^9-THC (3) + Oxy (3)	**	0.0093
	Δ^9-THC (3) vs. Oxycodone (3)	ns	0.1437
	Δ^9-THC (3) vs. Δ^9-THC (3) + Oxy (3)	*	0.036
	Oxycodone (3) vs. Δ^9-THC (3) + Oxy (3)	ns	0.9666
Recovery	Vehicle vs. Δ^9-THC (3)	ns	0.9968
	Vehicle vs. Oxycodone (3)	ns	0.3531
	Vehicle vs. Δ^9-THC (3) + Oxy (3)	ns	0.995
	Δ^9-THC (3) vs. Oxycodone (3)	ns	0.397
	Δ^9-THC (3) vs. Δ^9-THC (3) + Oxy (3)	ns	0.9762
	Oxycodone (3) vs. Δ^9-THC (3) + Oxy (3)	ns	0.391

Figure 5H – Circadian Activity Index Females

Type of test	Comparison	P-value summary	F, P-value
Two-way ANOVA	Time	****	F (2.505, 40.08) = 31.95, P<0.0001
	Treatment	ns	F (3, 16) = 1.591, P=0.2307
	Interaction	*	F (9,48) = 2.360, P=0.0269
Tukey's post-hoc			
Baseline	Vehicle vs. Δ^9-THC (3)	ns	0.9885
	Vehicle vs. Oxycodone (3)	ns	0.9877

Type of test	Comparison	P-value summary	F, P-value
	Vehicle vs. Δ^9-THC (3) + Oxy (3)	ns	0.9674
	Δ^9-THC (3) vs. Oxycodone (3)	ns	>0.9999
	Δ^9-THC (3) vs. Δ^9-THC (3) + Oxy (3)	ns	0.9973
	Oxycodone (3) vs. Δ^9-THC (3) + Oxy (3)	ns	0.9908
Dosing	Vehicle vs. Δ^9-THC (3)	ns	0.6514
	Vehicle vs. Oxycodone (3)	*	0.0275
	Vehicle vs. Δ^9-THC (3) + Oxy (3)	*	0.0151
	Δ^9-THC (3) vs. Oxycodone (3)	ns	0.0524
	Δ^9-THC (3) vs. Δ^9-THC (3) + Oxy (3)	**	0.0077
	Oxycodone (3) vs. Δ^9-THC (3) + Oxy (3)	ns	0.7014
Withdrawal	Vehicle vs. Δ^9-THC (3)	ns	0.9984
	Vehicle vs. Oxycodone (3)	ns	0.9376
	Vehicle vs. Δ^9-THC (3) + Oxy (3)	ns	0.9126
	Δ^9-THC (3) vs. Oxycodone (3)	ns	0.4898
	Δ^9-THC (3) vs. Δ^9-THC (3) + Oxy (3)	ns	0.0763
	Oxycodone (3) vs. Δ^9-THC (3) + Oxy (3)	ns	0.9666
Recovery	Vehicle vs. Δ^9-THC (3)	ns	>0.9999
	Vehicle vs. Oxycodone (3)	ns	>0.9999
	Vehicle vs. Δ^9-THC (3) + Oxy (3)	ns	0.7981
	Δ^9-THC (3) vs. Oxycodone (3)	ns	>0.9999
	Δ^9-THC (3) vs. Δ^9-THC (3) + Oxy (3)	ns	0.6732
	Oxycodone (3) vs. Δ^9-THC (3) + Oxy (3)	ns	0.6213

Supplemental Table 6. Statistics from Figure 6

Figure 6C – Male Oxycodone (3)

Type of test	Comparison	P-value summary	F, P-value
Two-way ANOVA	Time	ns	F (1,12) = 0.0264, P=0.8736
	Treatment	ns	F (1,12) = 2.435, P=0.1446
	Interaction	****	F (1,12) = 48.94, P<0.0001
Sidak post-hoc			
Pre-test	Vehicle vs. Oxycodone (3)	ns	0.3803
Post-test	Vehicle vs. Oxycodone (3)	***	0.0007

Figure 6D – Female Oxycodone (3)

Type of test	Comparison	P-value summary	F, P-value
Two-way ANOVA	Time	ns	F (1,14) = 0.01402, P=0.9074
	Treatment	ns	F (1,14) = 3.140, P=0.0982
	Interaction	****	F (1,14) = 81.60, P<0.0001
Sidak post-hoc			
Pre-test	Vehicle vs. Oxycodone (3)	ns	0.2123

Type of test	Comparison	P-value summary	F, P-value
Post-test	Vehicle vs. Oxycodone (3)	****	<0.0001

Figure 6E – Male Δ⁹-THC (3) + Oxycodone (1)

Type of test	Comparison	P-value summary	F, P-value
Two-way ANOVA	Time	ns	$F_{(1,12)} = 0.0023$, $P=0.8831$
	Treatment	ns	$F_{(1,12)} = 2.678$, $P=0.1277$
	Interaction	*	$F_{(1,12)} = 5.241$, $P=0.0421$
Sidak post-hoc			
Pre-test	Vehicle vs. Δ^9-THC (3) + Oxycodone (1)	ns	0.9658
Post-test	Vehicle vs. Δ^9-THC (3) + Oxycodone (1)	*	0.0328

Figure 6F – Female Δ^9-THC (3) + Oxycodone (1)

Type of test	Comparison	P-value summary	F, P-value
Two-way ANOVA	Time	ns	$F_{(1,10)} = 0.005$, $P=0.9427$
	Treatment	ns	$F_{(1,10)} = 2.501$, $P=0.1449$
	Interaction	****	$F_{(1,10)} = 6.729$, $P=0.0268$
Sidak post-hoc			
Pre-test	Vehicle vs. Δ^9-THC (3) + Oxycodone (1)	ns	0.9836
Post-test	Vehicle vs. Δ^9-THC (3) + Oxycodone (1)	*	0.0234

Figure 6G – Male Δ^9-THC (3) + Oxycodone (3)

Type of test	Comparison	P-value summary	F, P-value
Two-way ANOVA	Time	ns	$F_{(1,14)} = 0.003$, $P=0.8572$
	Treatment	**	$F_{(1,14)} = 11.36$, $P=0.0046$
	Interaction	****	$F_{(1,14)} = 41.26$, $P<0.0001$
Sidak post-hoc			
Pre-test	Vehicle vs. Δ^9-THC (3) + Oxycodone (3)	ns	0.9858
Post-test	Vehicle vs. Δ^9-THC (3) + Oxycodone (3)	****	<0.0001

Figure 6H – Female Δ^9-THC (3) + Oxycodone (3)

Type of test	Comparison	P-value summary	F, P-value
Two-way ANOVA	Time	ns	$F_{(1,14)} = 0.1671$, $P=0.6889$
	Treatment	ns	$F_{(1,14)} = 0.8393$, $P=0.3751$
	Interaction	*	$F_{(1,14)} = 8.366$, $P=0.0118$
Sidak post-hoc			
Pre-test	Vehicle vs. Δ^9-THC (3) + Oxycodone (3)	ns	0.5933
Post-test	Vehicle vs. Δ^9-THC (3) + Oxycodone (3)	*	0.044

Supplemental Table 7. Statistics from Figure 7

Figure 7A – Locomotor Sensitization Males

Type of test	Comparison	P-value summary	F, P-value
Two-way ANOVA	Time	*	F (1.578, 61.54) = 4.927, P=0.0161
	Treatment	****	F (5, 39) = 43.00, P<0.0001
	Interaction	****	F (10,78) = 4.995, P<0.0001
Tukey's post-hoc			
Day 1	Vehicle vs. Oxycodone (1)	ns	0.972
	Vehicle vs. Oxycodone (3)	**	0.0075
	Vehicle vs. Δ^9-THC (3)	ns	0.1219
	Vehicle vs. Oxycodone (1)+Δ^9-THC (3)	ns	0.7807
	Vehicle vs. Oxycodone (3)+Δ^9-THC (3)	*	0.0166
	Oxycodone (1) vs. Oxycodone (3)	*	0.0103
	Oxycodone (1) vs. Δ^9-THC (3)	*	0.0459
	Oxycodone (1) vs. Oxycodone (1)+Δ^9-THC (3)	ns	0.9636
	Oxycodone (1) vs. Oxycodone (3)+Δ^9-THC (3)	*	0.0265
	Oxycodone (3) vs. Δ^9-THC (3)	**	0.0031
	Oxycodone (3) vs. Oxycodone (1)+Δ^9-THC (3)	*	0.0202
	Oxycodone (3) vs. Oxycodone (3)+Δ^9-THC (3)	ns	0.7934
	Δ^9-THC (3) vs. Oxycodone (1)+Δ^9-THC (3)	ns	0.1286
	Δ^9-THC (3) vs. Oxycodone (3)+Δ^9-THC (3)	**	0.0047
	Oxycodone (1)+Δ^9-THC (3) vs. Oxycodone (3)+Δ^9-THC (3)	ns	0.0862
Day 2	Vehicle vs. Oxycodone (1)	ns	0.9372
	Vehicle vs. Oxycodone (3)	**	0.0016
	Vehicle vs. Δ^9-THC (3)	ns	0.2269
	Vehicle vs. Oxycodone (1)+Δ^9-THC (3)	ns	0.4254
	Vehicle vs. Oxycodone (3)+Δ^9-THC (3)	***	0.0002
	Oxycodone (1) vs. Oxycodone (3)	**	0.002
	Oxycodone (1) vs. Δ^9-THC (3)	ns	0.1176
	Oxycodone (1) vs. Oxycodone (1)+Δ^9-THC (3)	ns	0.7444
	Oxycodone (1) vs. Oxycodone (3)+Δ^9-THC (3)	***	0.0002
	Oxycodone (3) vs. Δ^9-THC (3)	**	0.001
	Oxycodone (3) vs. Oxycodone (1)+Δ^9-THC (3)	**	0.0044
	Oxycodone (3) vs. Oxycodone (3)+Δ^9-THC (3)	ns	0.9988

Type of test	Comparison	P-value summary	F, P-value
	Δ^9-THC (3) vs. Oxycodone (1)+Δ^9-THC (3)	ns	0.11
	Δ^9-THC (3) vs. Oxycodone (3)+Δ^9-THC (3)	***	0.0001
	Oxycodone (1)+Δ^9-THC (3) vs. Oxycodone (3)+Δ^9-THC (3)	***	0.0008
Day 3	Vehicle vs. Oxycodone (1)	ns	>0.9999
	Vehicle vs. Oxycodone (3)	***	0.0003
	Vehicle vs. Δ^9-THC (3)	*	0.0399
	Vehicle vs. Oxycodone (1)+Δ^9-THC (3)	ns	0.9992
	Vehicle vs. Oxycodone (3)+Δ^9-THC (3)	**	0.0083
	Oxycodone (1) vs. Oxycodone (3)	***	0.0005
	Oxycodone (1) vs. Δ^9-THC (3)	****	<0.0001
	Oxycodone (1) vs. Oxycodone (1)+Δ^9-THC (3)	ns	0.9997
	Oxycodone (1) vs. Oxycodone (3)+Δ^9-THC (3)	**	0.0089
	Oxycodone (3) vs. Δ^9-THC (3)	***	0.0002
	Oxycodone (3) vs. Oxycodone (1)+Δ^9-THC (3)	***	0.0002
	Oxycodone (3) vs. Oxycodone (3)+Δ^9-THC (3)	ns	0.955
	Δ^9-THC (3) vs. Oxycodone (1)+Δ^9-THC (3)	ns	0.0532
	Δ^9-THC (3) vs. Oxycodone (3)+Δ^9-THC (3)	**	0.0026
	Oxycodone (1)+Δ^9-THC (3) vs. Oxycodone (3)+Δ^9-THC (3)	**	0.007

Figure 7B – Locomotor Sensitization Females

Type of test	Comparison	P-value summary	F, P-value
Two-way ANOVA	Time	ns	F (1.944, 56.38) = 3.188, P=0.0501
	Treatment	****	F (5, 39) = 12.29, P<0.0001
	Interaction	ns	F (10,58) = 0.9856, P=0.4661
Tukey's post-hoc			
Day 1	Vehicle vs. Oxycodone (1)	ns	0.3059
	Vehicle vs. Oxycodone (3)	***	0.0003
	Vehicle vs. Δ^9-THC (3)	ns	0.6909
	Vehicle vs. Oxycodone (1)+Δ^9-THC (3)	ns	0.9933
	Vehicle vs. Oxycodone (3)+Δ^9-THC (3)	**	0.0028
	Oxycodone (1) vs. Oxycodone (3)	*	0.044
	Oxycodone (1) vs. Δ^9-THC (3)	ns	0.1359
	Oxycodone (1) vs. Oxycodone (1)+Δ^9-THC (3)	ns	0.6764

Type of test	Comparison	P-value summary	F, P-value
	Oxycodone (1) vs. Oxycodone (3)+Δ^9-THC (3)	*	0.0373
	Oxycodone (3) vs. Δ^9-THC (3)	***	0.0004
	Oxycodone (3) vs. Oxycodone (1)+Δ^9-THC (3)	**	0.002
	Oxycodone (3) vs. Oxycodone (3)+Δ^9-THC (3)	ns	0.9642
	Δ^9-THC (3) vs. Oxycodone (1)+Δ^9-THC (3)	ns	0.7059
	Δ^9-THC (3) vs. Oxycodone (3)+Δ^9-THC (3)	***	0.001
	Oxycodone (1)+Δ^9-THC (3) vs. Oxycodone (3)+Δ^9-THC (3)	**	0.004
Day 2	Vehicle vs. Oxycodone (1)	ns	0.1238
	Vehicle vs. Oxycodone (3)	*	0.0268
	Vehicle vs. Δ^9-THC (3)	ns	0.9963
	Vehicle vs. Oxycodone (1)+Δ^9-THC (3)	ns	0.5619
	Vehicle vs. Oxycodone (3)+Δ^9-THC (3)	**	0.0016
	Oxycodone (1) vs. Oxycodone (3)	ns	0.1583
	Oxycodone (1) vs. Δ^9-THC (3)	ns	0.0823
	Oxycodone (1) vs. Oxycodone (1)+Δ^9-THC (3)	ns	0.9715
	Oxycodone (1) vs. Oxycodone (3)+Δ^9-THC (3)	*	0.011
	Oxycodone (3) vs. Δ^9-THC (3)	*	0.0241
	Oxycodone (3) vs. Oxycodone (1)+Δ^9-THC (3)	ns	0.0963
	Oxycodone (3) vs. Oxycodone (3)+Δ^9-THC (3)	ns	0.9677
	Δ^9-THC (3) vs. Oxycodone (1)+Δ^9-THC (3)	ns	0.397
	Δ^9-THC (3) vs. Oxycodone (3)+Δ^9-THC (3)	**	0.0018
	Oxycodone (1)+Δ^9-THC (3) vs. Oxycodone (3)+Δ^9-THC (3)	**	0.0058
Day 3	Vehicle vs. Oxycodone (1)	ns	0.1898
	Vehicle vs. Oxycodone (3)	*	0.0131
	Vehicle vs. Δ^9-THC (3)	ns	0.46
	Vehicle vs. Oxycodone (1)+Δ^9-THC (3)	ns	0.5221
	Vehicle vs. Oxycodone (3)+Δ^9-THC (3)	*	0.0315
	Oxycodone (1) vs. Oxycodone (3)	ns	0.1528
	Oxycodone (1) vs. Δ^9-THC (3)	*	0.0332
	Oxycodone (1) vs. Oxycodone (1)+Δ^9-THC (3)	ns	0.9606
	Oxycodone (1) vs. Oxycodone (3)+Δ^9-THC (3)	ns	0.1223
	Oxycodone (3) vs. Δ^9-THC (3)	**	0.0053

Type of test	Comparison	P-value summary	F, P-value
	Oxycodone (3) vs. Oxycodone (1)+Δ^9-THC (3)	ns	0.069
	Oxycodone (3) vs. Oxycodone (3)+Δ^9-THC (3)	ns	0.8586
	Δ^9-THC (3) vs. Oxycodone (1)+Δ^9-THC (3)	ns	0.0654
	Δ^9-THC (3) vs. Oxycodone (3)+Δ^9-THC (3)	*	0.0178
	Oxycodone (1)+Δ^9-THC (3) vs. Oxycodone (3)+Δ^9-THC (3)	ns	0.0779

Supplemental Table 8. Statistics from Figure 8

Figure 8A – Δ9-THC (3 mg/kg) vs Vehicle

Type of test	Comparison	P-value summary	F, P-value
Paired t-test	All bins	ns	No bins reached raw p < 0.05

Figure 8B – Oxycodone (1 mg/kg) vs Vehicle

Type of test	Comparison	P-value summary	F, P-value
Paired t-test	1.5 min	*	t = 4.474, P=0.0110
Paired t-test	4.5 min	*	t = 4.603, P=0.0100
Paired t-test	7.5 min	*	t = 2.994, P=0.0402
Paired t-test	10.5 min	*	t = 2.780, P=0.0498
Paired t-test	13.5 min	*	t = 3.305, P=0.0298
Paired t-test	22.5 min	**	t = 5.048, P=0.0072
Paired t-test	25.5 min	**	t = 7.774, P=0.0015
Paired t-test	28.5 min	**	t = 6.578, P=0.0028
Paired t-test	31.5 min	*	t = 3.507, P=0.0247

Figure 8C – Oxycodone (3 mg/kg) vs Vehicle

Type of test	Comparison	P-value summary	F, P-value
Paired t-test	1.5 min	*	t = 3.542, P=0.0240
Paired t-test	4.5 min	**	t = 5.253, P=0.0063
Paired t-test	7.5 min	**	t = 6.828, P=0.0024
Paired t-test	10.5 min	**	t = 7.777, P=0.0015
Paired t-test	13.5 min	**	t = 5.388, P=0.0057
Paired t-test	16.5 min	**	t = 5.259, P=0.0063
Paired t-test	19.5 min	**	t = 4.826, P=0.0085
Paired t-test	22.5 min	*	t = 3.229, P=0.0320
Paired t-test	25.5 min	*	t = 3.168, P=0.0339
Paired t-test	28.5 min	*	t = 3.034, P=0.0386
Paired t-test	31.5 min	*	t = 3.666, P=0.0215
Paired t-test	40.5 min	*	t = 3.258, P=0.0311
Paired t-test	43.5 min	*	t = 4.269, P=0.0130
Paired t-test	46.5 min	**	t = 5.179, P=0.0066
Paired t-test	49.5 min	*	t = 3.947, P=0.0169
Paired t-test	58.5 min	*	t = 3.201, P=0.0329

Figure 8D – Oxycodone (1 mg/kg) + Δ9-THC (3 mg/kg) vs Vehicle

Type of test	Comparison	P-value summary	F, P-value
Paired t-test	4.5 min	**	t = 7.344, P=0.0018
Paired t-test	10.5 min	*	t = 3.308, P=0.0297
Paired t-test	13.5 min	*	t = 4.339, P=0.0123
Paired t-test	16.5 min	*	t = 4.213, P=0.0135
Paired t-test	22.5 min	**	t = 6.837, P=0.0024
Paired t-test	25.5 min	**	t = 8.120, P=0.0013
Paired t-test	28.5 min	**	t = 5.444, P=0.0055
Paired t-test	31.5 min	*	t = 3.205, P=0.0327
Paired t-test	34.5 min	**	t = 6.175, P=0.0035
Paired t-test	37.5 min	**	t = 7.692, P=0.0015
Paired t-test	40.5 min	*	t = 4.522, P=0.0106
Paired t-test	43.5 min	***	t = 10.411, P=0.0005
Paired t-test	49.5 min	*	t = 2.908, P=0.0438

Figure 8E – Oxycodone (1 mg/kg) + Δ9-THC (3 mg/kg) vs Oxycodone (1 mg/kg)

Type of test	Comparison	P-value summary	F, P-value
Paired t-test	16.5 min	*	t = 2.936, P=0.0425
Paired t-test	25.5 min	*	t = 3.267, P=0.0309
Paired t-test	28.5 min	*	t = 2.879, P=0.0451
Paired t-test	34.5 min	*	t = 4.157, P=0.0142
Paired t-test	37.5 min	*	t = 3.675, P=0.0213
Paired t-test	43.5 min	*	t = 3.492, P=0.0251

Figure 8F – 0–10 min

Type of test	Comparison	P-value summary	F, P-value
Repeated-measures ANOVA	All groups	****	F (4, 16) = 14.462, P=0.0000
Paired post-hoc (Holm)	Vehicle vs. Oxycodone (3)	*	0.0275
Paired post-hoc (Holm)	Oxycodone (3) vs. Δ9-THC (3)	*	0.0275

Figure 8F – 10–20 min

Type of test	Comparison	P-value summary	F, P-value
Repeated-measures ANOVA	All groups	***	F (4, 16) = 10.695, P=0.0002
Paired post-hoc (Holm)	Vehicle vs. Oxycodone (3)	*	0.0229
Paired post-hoc (Holm)	Δ9-THC (3) vs. Oxycodone (1)+Δ9-THC (3)	*	0.0282

Figure 8F – 20–30 min

Type of test	Comparison	P-value summary	F, P-value
Repeated-measures ANOVA	All groups	**	F (4, 16) = 5.501, P=0.0056
Paired post-hoc (Holm)	Vehicle vs. Oxycodone (1)	*	0.0164
Paired post-hoc (Holm)	Vehicle vs. Oxycodone (1)+Δ9-THC (3)	*	0.0156

Figure 8F – 30–40 min

Type of test	Comparison	P-value summary	F, P-value
Repeated-measures ANOVA	All groups	*	F (4, 16) = 3.412, P=0.0337
Paired post-hoc (Holm)	Vehicle vs. Oxycodone (1)+Δ9-THC (3)	*	0.0378

Figure 8F – 40–50 min

Type of test	Comparison	P-value summary	F, P-value
Repeated-measures ANOVA	All groups	*	F (4, 16) = 3.596, P=0.0283
Paired post-hoc (Holm)	Vehicle vs. Oxycodone (3)	*	0.0469

Figure 8F – 50–60 min

Type of test	Comparison	P-value summary	F, P-value
Repeated-measures ANOVA	All groups	ns	F (4, 16) = 2.194, P=0.1160
Paired post-hoc (Holm)	No significant comparisons	–	–

Figure 8F – Vehicle / Oxycodone (1) / Δ9-THC (3) / Oxycodone (1) + Δ9-THC (3)

Type of test	Comparison	P-value summary	F, P-value
Oxycodone main	0–10 min	*	F (1, 4) = 16.225, P=0.0158
Oxy × Δ9-THC interaction	0–10 min	ns	F (1, 4) = 3.175, P=0.1494
Oxycodone main	10–20 min	*	F (1, 4) = 14.910, P=0.0181
Oxy × Δ9-THC interaction	10–20 min	*	F (1, 4) = 20.365, P=0.0107
Oxycodone main	20–30 min	**	F (1, 4) = 24.113, P=0.0080
Oxy × Δ9-THC interaction	20–30 min	ns	F (1, 4) = 0.920, P=0.3919
Oxy × Δ9-THC interaction	30–40 min	ns	F (1, 4) = 0.002, P=0.9630

Figure 8F – Planned contrasts

Type of test	Comparison	P-value summary	F, P-value
Paired t (Holm)	Oxycodone (1)+Δ9-THC (3) vs. Vehicle (0–10 min)	*	0.0411
Paired t (Holm)	Oxycodone (1)+Δ9-THC (3) vs. Vehicle (20–30 min)	**	0.0047
Paired t (Holm)	Oxycodone (1)+Δ9-THC (3) vs. Vehicle (30–40 min)	*	0.0113
Paired t (Holm)	Oxycodone (1)+Δ9-THC (3) vs. Vehicle (40–50 min)	*	0.0195
